# *RP1* Dominant p.Ser740* Pathogenic Variant in 20 Knowingly Unrelated Families Affected by Rod–Cone Dystrophy: Potential Founder Effect in Western Sicily

**DOI:** 10.3390/medicina60020254

**Published:** 2024-02-01

**Authors:** Fabiana D’Esposito, Viviana Randazzo, Maria Igea Vega, Gabriella Esposito, Paolo Enrico Maltese, Salvatore Torregrossa, Paola Scibetta, Florinda Listì, Caterina Gagliano, Lucia Scalia, Antonino Pioppo, Antonio Marino, Marco Piergentili, Emanuele Malvone, Tiziana Fioretti, Angela Vitrano, Maria Piccione, Teresio Avitabile, Francesco Salvatore, Matteo Bertelli, Ciro Costagliola, Maria Francesca Cordeiro, Aurelio Maggio, Elena D’Alcamo

**Affiliations:** 1 Imperial College Ophthalmic Research Group (ICORG) Unit, Imperial College, London SW7 2AZ, UK; m.cordeiro@imperial.ac.uk; 2 Eye Clinic, Department of Neurosciences, Reproductive Sciences and Dentistry, University of Naples Federico II, 80100 Naples, Italy; emanuelemalvone@gmail.com (E.M.); ciro.costagliola@unina.it (C.C.); 3 Genofta s.r.l., Sant’Agnello, 80065 Naples, Italy; 4 Eye Clinic, AOOR Villa Sofia-Cervello, 90100 Palermo, Italy; vivianarandazzo86@gmail.com (V.R.); torregrossa.s@gmail.com (S.T.); paolascibetta@tiscali.it (P.S.); 5 Department of Genetics, Oncohaematology and Rare Diseases, AOOR Villa Sofia-Cervello, 90100 Palermo, Italy; igeavega@gmail.com (M.I.V.); f.listi72@gmail.com (F.L.); angelavitranos4@gmail.com (A.V.); maria.piccione@unipa.it (M.P.); aurelio.maggio@villasofia.it (A.M.); e.dalcamo@villasofia.it (E.D.); 6 Department of Molecular Medicine and Medical Biotechnologies, University of Naples Federico II, 80100 Naples, Italy; gabriella.esposito@unina.it (G.E.); salvator@unina.it (F.S.); 7 CEINGE-Advanced Biotechnologies Franco Salvatore, 80100 Naples, Italy; fioretti@ceinge.unina.it; 8 MAGI’S Lab s.r.l., 38068 Rovereto, Italy; paolo.maltese@assomagi.org; 9 Department of Medicine and Surgery, School of Medicine, Kore University of Enna, 94100 Enna, Italy; caterina_gagliano@hotmail.com; 10 Eye Clinic, Catania University, Policlinico “Rodolico”-San Marco, 95100 Catania, Italy; scalia-lucia@virgilio.it (L.S.); t.avitabile@unict.it (T.A.); 11 Eye Clinic, ARNAS Civico, 90100 Palermo, Italy; ninopioppo@gmail.com; 12 Department of Ophthalmology, Garibaldi Hospital, 95100 Catania, Italy; amarino.oculista@yahoo.it; 13 Department of Ophthalmology, Careggi Teaching Hospital, 50100 Florence, Italy; mpiergent@gmail.com; 14 MAGI Euregio, 39100 Bolzano, Italy; matteo.bertelli@assomagi.org

**Keywords:** rod–cone dystrophy, retinitis pigmentosa, *RP1* gene, founder effect, inherited retinal dystrophies, founder mutation

## Abstract

*Background and Objectives*. Retinitis pigmentosa (RP) is the most common inherited rod–cone dystrophy (RCD), resulting in nyctalopia, progressive visual field, and visual acuity decay in the late stages. The autosomal dominant form (ADRP) accounts for about 20% of RPs. Among the over 30 genes found to date related to ADRP, *RP1* pathogenic variants have been identified in 5–10% of cases. In a cohort of RCD patients from the Palermo province on the island of Sicily, we identified a prevalent nonsense variant in *RP1*, which was associated with ADRP. The objective of our study was to analyse the clinical and molecular data of this patient cohort and to evaluate the potential presence of a founder effect. *Materials and Methods*. From 2005 to January 2023, 84 probands originating from Western Sicily (Italy) with a diagnosis of RCD or RP and their relatives underwent deep phenotyping, which was performed in various Italian clinical institutions. Molecular characterisation of patients and familial segregation of pathogenic variants were carried out in different laboratories using Sanger and/or next-generation sequencing (NGS). *Results.* Among 84 probands with RCD/RP, we found 28 heterozygotes for the *RP1* variant c.2219C>G, p.Ser740* ((NM_006269.2)*, which was therefore significantly prevalent in this patient cohort. After a careful interview process, we ascertained that some of these patients shared the same pedigree. Therefore, we were ultimately able to define 20 independent family groups with no traceable consanguinity. Lastly, analysis of clinical data showed, in our patients, that the p.Ser740* nonsense variant was often associated with a late-onset and relatively mild phenotype. *Conclusions*. The high prevalence of the p.Ser740* variant in ADRP patients from Western Sicily suggests the presence of a founder effect, which has useful implications for the molecular diagnosis of RCD in patients coming from this Italian region. This variant can be primarily searched for in RP-affected subjects displaying compatible modes of transmission and phenotypes, with an advantage in terms of the required costs and time for analysis. Moreover, given its high prevalence, the *RP1* p.Ser740* variant could represent a potential candidate for the development of therapeutic strategies based on gene editing or translational read-through therapy for suppression of nonsense variants.

## 1. Introduction

Inherited retinal dystrophies (IRDs) include a spectrum of retinal diseases characterised by genetically determined dysfunction and progressive degeneration of the retina. Retinitis pigmentosa (RP) traditionally defines a subgroup of IRDs in which rods are primarily affected with respect to cones (rod–cone dystrophies, RCDs), with an estimated prevalence of 1/3000–1/5000 [1,2]. RCD-affected patients experience a visual impairment characterised by nyctalopia, progressive concentric reduction in the visual field, and decay in visual acuity in the advanced stages. Classic signs include optic disc pallor, attenuated retinal vessels, and diffuse pigmentary changes in the retina. An abnormal or unrecordable electroretinogram (ERG), in which the scotopic component is altered more severely and earlier than the photopic one, is the hallmark of this type of retinal dystrophy. Age at onset can be very variable, usually within the first two decades of life, but it is not uncommon to appear later [3]. In RP, all modes of transmission can be seen, with different percentages in the general population: Autosomal dominant (ADRP) 15–25%, autosomal recessive (ARRP) 5–20%, X-linked (XLRP) 5–15%, and rarely mitochondrial or digenic. Forty to fifty percent of patients are sporadic (simplex RP), with an unremarkable family history for the condition [4,5,6].

Nonsyndromic RP is genetically highly heterogeneous. To date, about 30 genes have been associated with ADRP, 66 with ARRP, and three with the XLRP form, in addition to several loci [7,8]. However, in a consistent number of patients, the causative molecular defect cannot be identified, leading to the conclusion that other genes have yet to be characterised, or that different mechanisms can play a pathogenic role.

*RP1* (OMIM #603937) was identified in 1999 as the gene underlying the RP9 locus-linked form of RP. It is located on chromosome 8q12.1 and encompasses four exons, of which three are coding [9,10]. It encodes an oxygen-regulated photoreceptor protein that is localised in the connecting cilia of both cones and rods and has a role in the transport of proteins between the inner and outer segments of photoreceptors, in cilial structure maintenance, and in the stabilisation of disc membranes in the outer segments [11].

About 500 unique genomic variants have been reported in the *RP1*-specific variation database (LOVD, https://databases.lovd.nl/shared/genes/RP1, accessed on 15 November 2023), mostly represented by small nucleotide variants (SNVs) that cause missense, nonsense, frameshift, and splicing changes. Interestingly, *RP1* pathogenic variants can have a dominant or recessive effect [12,13,14]. In particular, pathogenic variants leading to truncated proteins show a peculiar correlation between the location of the premature stop codon (PTC) and the pattern of inheritance. Usually, truncation variants located in the middle portion of the RP1 protein contribute to ADRP, while variants leading to PTC in the N- and C-terminals are associated with ARRP. Various studies approximated the location of the middle portion of the RP1 protein from amino acid p.661 to p.917 [14]. In agreement, in this protein region, the PTC arising from the c.2029C>T (p.Arg677*) nonsense variant is located, representing the most reported dominant pathogenic variant in *RP1* [15,16]. In this scenario, the evidence that patients heterozygous for deletions removing most sequences of the *RP1* gene are unaffected strongly indicates that *RP1* is not a haploinsufficient gene and supports a dominant-negative effect for truncation variants associated with ADRP [17].

Variants in the *RP1* gene underlie 5–10% of ADRP cases, but this prevalence varies between studies and populations [5,15]. Incomplete penetrance and variable expressivity are also documented for *RP1*-related ADRP [10]. Indeed, individuals affected by dominantly inherited *RP1* variants usually display a classic phenotype of RP and suffer from nyctalopia and progressive visual field concentric reduction. In contrast, most patients preserve a relatively acceptable visual acuity and reduced—but still present—residual central visual field for many years. As in other forms of RP, early cataracts, cystoid macular oedema (CME), and pre-retinal fibrosis can complicate the visual outcome in some patients.

This study originates from the molecular characterisation of the genetic causes of RCD/RP in a cohort of patients sharing the same geographic origin: Western Sicily. As a consequence of the identification of a recurrent pathogenic variant in the *RP1* gene of ADRP patients, we aimed to evaluate the potential presence of a founder effect in this Italian region.

## 2. Materials and Methods

Between 2005 and 2023, 84 unrelated probands (50% males, 50% females) who were attending the medical retina clinics participating in this study obtained a clinical and instrumental diagnosis of RCD or RP and underwent genetic testing. The participating clinical centres were the following: CTO Unit and Cervello Hospital, AOOR Villa Sofia-Cervello, Palermo; UOC Oculistica, ARNAS Civico, Palermo; Eye Clinic, Catania University, Policlinico “Rodolico”—San Marco, Catania; Department of Ophthalmology, Garibaldi Hospital, Catania; and Eye Clinic, University of Naples, “Federico II”. Genetic testing was performed in the laboratories of the following institutions: CEINGE-Biotecnologie Avanzate Franco Salvatore, Naples; MAGI’s Lab, Rovereto/MAGI Euregio, Bolzano; and AOOR Villa Sofia-Cervello, Dipartimento di Genetica Oncoematologia e Malattie Rare, Palermo.

Patients shared the same geographic origin: The Palermo province in Western Sicily, Italy. This is an area of around 5000 sqKm with approximately 1,275,000 inhabitants.

All patients or their guardians provided written informed consent to the use of their anonymised genetic data for research purposes and scientific publications. The present study was conducted adhering to the tenets of the Declaration of Helsinki.

### 2.1. Clinical and Instrumental Evaluation

Patients were diagnosed at different clinical institutions with various equipment. In most cases, we could obtain comprehensive ophthalmic examinations, including best-corrected visual acuity (BCVA), intraocular pressure (IOP) measurement (noncontact tonometer), slit-lamp anterior and posterior segment biomicroscopy, colour fundus photography, autofluorescence, OCT and OCT angiography (the latter in a limited number of patients), stationary perimetry tests, and dark- and light-adapted ERGs performed according to ISCEV standards. When not performed personally by any of the authors of this study, provided documentation was evaluated to establish or confirm a diagnosis.

### 2.2. Transmission Mode Definition

Genealogies were established through interviews with patients and their family members. In particular, the surnames of the maternal branches were recorded in order to unmask less obvious common origins. When no positive family history was reported, patients were defined as sporadic. A consistent number of the probands’ relatives who reported themselves as unaffected actually carried the *RP1* p.Ser740* pathogenic variant. In these cases, clinical and instrumental testing were carried out, and the pathologic phenotype was defined after molecular diagnosis. Pedigrees and their captions are shown in the Supplementary Material (SM 1–21).

### 2.3. Genetic Analytic Strategy

Patients of the selected cohort were analysed in different centres and with different molecular methodologies depending on the year of examination.

The *RP1* p.Ser740* variant was identified in two probands (#10 and #26) through Sanger sequencing of the following candidate genes for ADRP at CEINGE (Naples, Italy) in 2005: *RHO*, *RP1*, and *PRPH2*. All other patients underwent NGS analysis. Patients #2, 4, 6, 7, 11, 14, 15, 16, 20, 27, and 28 were tested at AOOR Villa Sofia-Cervello (Palermo, Italy) on a panel of 70 RP-related genes, while patients #1, 3, 5, 8, 9, 12, 13, 17, 18, 19, 21, 22, 23, 24, and 25 were tested at MAGI’S in Bolzano (Italy) on a panel of 73 genes (Supplementary Material, SM22). The choice of genes that were in the panels reflected data emerging from both the literature and each laboratory case series. Potentially pathogenic variants were validated through Sanger sequencing.

All variants were searched in the Human Gene Mutation Database, professional version 2023.1 (https://my.qiagendigitalinsights.com/bbp/view/hgmd/, accessed on 1 September 2023); dbSNP (https://www.ncbi.nlm.nih.gov/snp/, accessed on 1 September 2023); ClinVar (https://www.ncbi.nlm.nih.gov/clinvar/, accessed on 1 September 2023); LOVD, Leiden Open Variation Database (https://www.lovd.nl/, accessed on 1 September 2023); and gnomAD (https://gnomad.broadinstitute.org/, accessed on 1 September 2023). The pathogenic role of the identified variants was predicted using the bioinformatic prediction tool Varsome (https://varsome.com/, accessed on 1 September 2023), which classifies variants according to the American College of Medical Genetics and Genomics standards and guidelines [18].

Segregation analysis was also performed for informative and available family members, often following specific requests for the definition of pre-symptomatic status. Only variants confirmed to be pathogenic or likely pathogenic through in silico tools and familial segregation were reported.

### 2.4. Statistical Evaluation

Descriptive statistics were presented as medians (interquartile range (IQR)) and percentages. The proportion of RCD/RP patients with different transmission modes as well as the prevalence of genes associated with AD forms in the general population were estimated by applying the definition of a proportion. The average value of the percentage variation range, as reported in the literature [6,7], was calculated and compared with the percentages of the same transmission modes and ADRP-causative genes in our cohort of patients.

Differences in percentages regarding the modes of transmission of CRD/RP between our cohort of patients and the general population, as well as in the causative genes for the AD form, were evaluated using a two-sided z-test for the comparison of two samples at the 5% significance level.

## 3. Results

### 3.1. Molecular Data Analysis

In our cohort of 84 probands with a clinical diagnosis of RP or RCD, family history suggested different patterns of Mendelian inheritance: 35 AD (41%), 20 AR (24%), and 4 XL (5%) putative transmission modes. Twenty-five probands (30%) did not report any family history and were therefore defined as sporadic (Table 1).

Disease-causing variants were found in the following genes, which are all described in the literature as RP related [7,8].

Among the 35 ADRP patients, 26 had heterozygous variants in *RP1* c.2219C>G, p.Ser740* ((NM_006269.2), being the only variant identified in all of them), 5 in *RHO*, 2 in *FSCN2*, and 1 for each in *TOPORS* and *RP9.*

Among the 20 ARRP patients, 7 probands were affected by biallelic variants in *USH2A*; 1 patient each had mutations in *PROM1*, *PCARE*, *PDE6B*, *CDHR1*, *IMPG2*, *MERTK*, *TULP1*, *EYS*, and *PDE6A*; in 4 patients, the molecular analysis was inconclusive. Three patients with XLRP carried *RPGR* variants, and one patient was hemizygous for an RP2 mutation. Molecular analysis performed for the 25 sporadic patients was inconclusive in 18 cases; 2 patients were heterozygous for the *RP1* p.Ser740* variant, while the remaining patients were carrying pathogenic variants in *SNRNP200*, *CRB*, *PROM1*, *EYS*, and *USH2A*. Clinical and molecular details of patients carrying variants in genes other than RP1 in this cohort are the subject of a further study, still in progress. Results of genetic analysis were considered conclusive only when sequence variants were unambiguously verified using in silico tools, coupled with consistent familial segregation.

Overall, the pathogenic *RP1* nonsense variant c.2219C>G (p.Ser740*) was identified in the heterozygous state in 28 patients with *RP1*-related RCD, i.e., 26 with AD pedigrees and 2 sporadic, representing 33.3% of all the examined probands, in contrast with the expected 5–10%. Therefore, compared to the general population, this prevalence results in a statistically significant change in the percentages of inheritance modes (Table 1) as well as in the molecular epidemiology of *RP1*-related RP/RCD in this Sicilian cohort of patients (*p* = 0.0073).

The 28 probands found to carry this variant independently accessed molecular genetic testing. After careful interviewing, we identified a shared pedigree for a proportion of them, identifying a total of 20 independent family groups with no traceable consanguinity. An examination of accessory variants identified in other genes via multigene NGS analysis did not reveal any other shared variants, with the exception of a benign *CA4* variant (c.700G>A, p.Val234Ile) that was present in both family #1 and family #18 (Table 2); the two families would not have otherwise been connected to a common ancestor through interviewing. Among the 20 pedigrees, 4 included more than one of our probands with a total of 12 patients, while for the remaining, we could not trace any consanguinity through focused interviewing (Supplementary Material, Pedigrees).

Autosomal dominant inheritance was confirmed in all the pedigrees of the patients carrying the *RP1* Ser740* variant, except the 2 sporadic cases (patients #21, #24), who did not report any affected ascendant or sibling and did not provide availability for family members phenotyping and/or genotyping. In one exceptionally large pedigree (Pedigree CLB, Supplementary Material), we also identified an elevated degree of consanguinity, but none of the patients, either probands or tested affected family members, was homozygous for the variant, nor were there particularly severe cases reported from family history.

In many cases, due to the relatively mild form of RP caused by the described variant, family members initially reported as nonaffected were then found to display signs of RP on clinical and instrumental evaluation. Due to this and the late onset of symptoms and signs, the definition of the relatives’ status was considered only partially reliable when based exclusively on the patients’ interviews.

### 3.2. Phenotype–Genotype Correlation in RP1-Associated ADRP

The phenotype related to *RP1* p.Ser740* was consistent with the diagnosis of RCD or RP in all patients. Indeed, all patients experienced nyctalopia as the first sign, with a reported median age at onset of 35 years (IQR 26.7–41.3) (Table 2). Visual field concentric reduction, resulting in the typical “tunnel vision”, and nyctalopia, were present in all patients.

**Figure 1:**
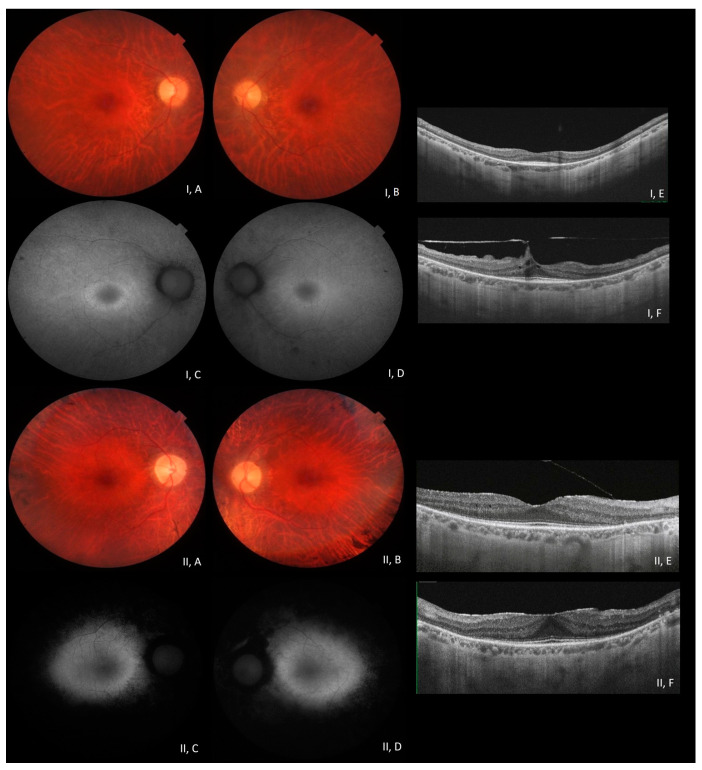
Multimodal imaging in patients #28 (**I**) and #9 (**II**) (clinical and genetic details in Table 2). Posterior pole fundus colour pictures ((**I**, **A**,**B**) and (**II**, **A**,**B**)) showing pale optic disc, attenuated vessels, and diffuse retinal thinning, relatively sparing the macular region. Posterior pole blue autofluorescence (BAF) showing the macular hyper-autofluorescent ring, typical in RP (**I**, **C**,**D**); in patient #9, the more advanced stage of the disease appears with extensive hypoautofluorence surrounding the macular region (**II**, **C**,**D**). OCT ((**I**, **E**,**F**) and (**II**, **E**,**F**)) shows a portion of the subfoveal preserved ellipsoid zone, with diffuse disruption elsewhere, preretinal fibrosis, and vitreomacular traction in patient #28 (OS (**I,F**)).

At fundus examination, the classic signs of retinitis pigmentosa (pale optic disc, vessel attenuation, and bone-spicule-shaped pigmented deposits) were present, and imaging was consistent with the diagnosis in all patients (Figure 1). In some patients, early cataracts, pre-retinal fibrosis, and cystoid macular oedema (CME) were reported complications (Table 2), consistent with the condition. In particular, CME was present in four patients who showed a good response to topical carbonic anhydrase inhibitors. Most patients underwent electrodiagnostic testing, displaying severely reduced or unrecordable responses under scotopic conditions and reduced or absent responses under photopic conditions in the more advanced stages.

A summary of clinical and genetic data is shown in Table 2. Multimodal imaging of selected patients (#9 and #28) is also shown in Figure 1.

The phenotype appears relatively mild in most affected patients, with preservation of central vision until later decades of age. Exceptions were two patients who had undergone unconventional invasive treatments in the past, with complications such as endophthalmitis (patient #26 OS) and laser-induced damage (patient #2 OU). In several family members, clinical diagnosis followed the molecular diagnosis, even in older patients. This is expected, given the relatively mild phenotype associated with the p.Ser740* variant.

To explain the more severe presentation in some patients (excluding the aforementioned patients #2 and #26), we examined available molecular data regarding potential additional effects of accessory variants in the same or in other genes (Table 2). All additional variants were evaluated using in silico tools, with results reported in the table. Significantly, patient #24, who showed the most severe phenotype, especially in terms of age at onset, was found to be compound heterozygous for a very rare intronic variant (c.788-12A>G) in *RP1*, which was predicted to be a variant of uncertain significance (VUS).

Although some expected differences in the phenotype in terms of age at onset and severity have been noted, the inter- and intrafamilial variability is low overall. Patients generally report the onset of symptoms (nyctalopia and visual field concentric reduction) at adult ages, with a slow progression. Unsurprisingly, when a positive family history was present, symptoms appeared to be recognised and reported earlier by the patients; they were better recognised when older family members were affected by the condition, but there was no substantial intrafamilial difference in the phenotype.

## 4. Discussion

In a cohort of patients affected by RP/RCD originating from Western Sicily, we found a statistically significant (*p*-value = 0.0073) high prevalence of the *RP1* c.2219C>G (p.Ser740*) pathogenic variant, proven to be causative in 33.3% of the 84 examined probands and 74% of the 25 clearly AD pedigrees. This considerable prevalence causes a statistically significant imbalance towards the autosomal dominant mode of transmission of RP in the described cohort of patients.

According to the classification proposed by Chen et al. (2010) [12], this sequence variant would fall under class II, which includes nonsense-mediated decay (NMD)-insensitive truncations with a dominant-negative effect leading to RP.

Interestingly, the high prevalence of this variant in the Sicilian population has been recently mentioned by Karali et al. [19], with the report of 13 cases from nine apparently unrelated Sicilian families; however, this was only a general observation. The p.Ser740* variant in *RP1* was first identified in a male patient belonging to a large cohort recruited in London at the Moorfields Eye Hospital [20], but with no specification about his geographic origin. Again, the variant has been reported in a study conducted on 74 families from the United Arab Emirates [21], where it is generically mentioned that one of the probands was of Italian origin, but without a specific correlation between the identified variants and the ethnic origin of single patients. We can speculate on different hypotheses, for instance, that this latter patient was of Sicilian origin or vice versa that the founder variant is of an Arab origin, considering the very consistent presence of the Arab population in Western Sicily through the past centuries [22]. It is also possible that in these sporadically described cases, the variant occurred spontaneously, without any link to the Sicilian population.

The statistically significant prevalence of a genetic variant in a specific population [23] can either be the result of the presence of a founder effect (a variant originating from a single individual being diffused through many descendants across generations in a specific population) [24] or reflect the existence of a hotspot for mutation within a gene (an unstable DNA base pair, prone to mutation) [25]. We would exclude this second possibility, as the higher prevalence would not be confined to such a specific geographic area but would have been reported in other studies describing the molecular characterisation of large cohorts of patients [26]. Considering this, we did not perform a haplotype analysis showing co-segregation of specific markers with the variant, as the striking prevalence in this localised population makes the founder effect the most likely scenario.

At the same time, we would exclude “bottleneck effects” of the specific geographic localisation of this dominant variant as the result of other events, such as confinement, as Western Sicily has a wide territory and is a well-connected area, and/or a specific selection related to other genetically related conditions, as the RP1 protein is essentially expressed in the retina.

Two patients in the described cohort did not report a family history and appeared to be sporadic. Their ascendants were not available for phenotyping or genotyping. It is worth mentioning that in some cultural contexts and older generations, visual disability could be considered something to hide. In the characterisation of family members related to this study, it was not uncommon that we came across individuals who were shown to be affected based on both clinical and genetic investigations but denied the condition and reported themselves as unaffected. In addition, nonpaternity is a possible situation to be considered [27,28].

The widespread nature of this variant is likely to be related to the relatively mild phenotype and to the age of onset; being after the primary reproductive age, patients are therefore not informed in time about the transmission risk or the possible prevention with prenatal diagnosis or preimplantation genetic diagnosis [29].

Inherited retinal dystrophies still represent an important cause of visual impairment and blindness, with great efforts being made by the scientific community towards the characterisation of pathogenic mechanisms and ultimately therapeutic strategies [30]. Given the high genetic and phenotypic heterogeneity of IRDs, molecular diagnosis is crucial for the correct definition of each patient’s condition, for familial genetic counselling, for a better understanding of pathogenic mechanisms, and for creating datasets of patients based on the phenotype-related genetic defect, rather than exclusively on the phenotype itself, especially in the perspective of developing potential innovative therapies [31].

In the described form of autosomal dominant rod–cone dystrophy, or retinitis pigmentosa, the phenotype is relatively mild with a generally late onset, leading to a widespread diffusion of the condition in the studied population, where the *RP1* p.Ser740* pathogenic variant is most likely present as the result of a founder effect. The described variant is significantly prevalent in the Palermo Province of Western Sicily, thereby giving the possibility of modifying the diagnostic strategy in this population. Indeed, whenever a compatible phenotype and mode of transmission are identified, the screening for this specific variant could precede NGS testing, presenting great advantages in terms of resource usage and the time required to analyse a significant proportion of RP patients of Western Sicily ancestry, especially since NGS is a diagnostic strategy of limited availability in the region.

Finally, we identified a genetically homogeneous population affected by a condition caused by a nonsense variant.

Nonsense variants determine the generation of a stop codon (premature termination codon, PTC) that, in the translation process, results in the generation of a shorter and often nonfunctional protein. The nonsense-mediated decay mechanism avoids the accumulation of misfolded proteins.

Translational read-through therapy is a recently studied approach aiming at the specific targeting of nonsense mutations. It is essentially based on the effect of specific molecules, known as translational read-through-inducing drugs (TRIDs), which, through a ribosome-binding mechanism, can suppress a nonsense variant, therefore allowing the synthesis of a full-length protein [32,33].

## 5. Conclusions

We identified a significantly prevalent pathogenic variant in the *RP1* gene causing RP/RCD in a particular area in Western Sicily, which was very likely to be the result of a founder effect. This finding might represent an advantage for genetic diagnostic strategies.

The cohort of patients studied here is particularly large, and the majority have several affected family members. In this scenario, we believe that the identification of such a large and genetically uniform population of patients affected by a dominant nonsense-variant-related condition can be of great interest to the scientific community for the development of potential diagnostic and/or therapeutic approaches.

## Figures and Tables

**Table 1:** Statistical evaluation of transmission mode.

Transmission Mode	RP General Population Transmission Mode (%) ^#^	RP General Population Transmission Mode (Average %)	Described RP Cohort Transmission Mode (%)	*p*-Value
ADRP	15–25%	20.0	41.0	0.0181 *
ARRP	5–20%	12.5	24.0	0.1284
XLRP	5–15%	10.0	5.0	0.1465
SPORADIC RP	40–50%	45.0	30.0	0.0257 *

^#^ References [6,7]; * significant *p*-value.

**Table 2:** Clinical and genetic data of patients carrying the *RP1* Ser740* variant.

Pt ID	Sex	Age of Onset (Years)	Duration of Symptoms at Observation (Years)	BCVA; Associated Signs	Family History	Traceable Consanguinity	Other Variants (ACMG)
1	F	40	6	OD 20/20–OS 20/20; CME	+	ND	*CA4*: c.700G>A (B)
2	M	30	35	OD HM–OS HM	+	ND	*USH2A*: c.12112A>G (VUS)
3	F	26	39	OD 20/40–OS 20/40; PE OU	+	ND	ND
4	F	21	18	OD 20/25–OS 20/25; PE OU	+	Family CLB	*CNGB1*: c.3122G>A (VUS)
5	F	45	20	OD 20/25–OS 20/30; PE OU	+	Family CR	ND
6	M	27	<1	OD 20/20–OS 20/20; R-CME OU	+	Family CLB	ND—*RP1* sequencing
7	M	50	25	OD 20/100–OS 20/100	+	Family CLB	ND
8	F	40	10	OD 20/25–OS 20/30; PE OU	+	Family CPL	ND
9	M	40	23	OD 20/25–OS 20/30; CAT/PRF OU	+	Family CLB	ND
10	F	35	35	OD 20/40–OS 20/100; PE/CD/CME OU	+	ND	ND—*RP1* sequencing
11	F	55	1	OD 20/30–OS 20/20	+	ND	ND
12	M	26	7	OD 20/20–OS 20/20	+	ND	*OFD1*: c.815A>G (LB/VUS)
13	F	45	1	OD 20/25–OS 20/25; CAT OU; PRF OS	+	ND	ND
14	F	31	8	OD 20/25–OS 20/30	+	ND	ND
15	M	26	11	OD 20/100–OS 20/50	+	ND	*ABCA4*: c.6089G>A (P)
16	M	20	24	OD 20/20–OS 20/30; S-IOLO	+	ND	*PROM1*: c.652C>T (P)
17	M	35	5	OD20/25–OS 20/30; CME OU	+	Family CPL	ND
18	M	50	23	OD 20/70–OS 20/70; PE OU	+	ND	*IMPDH1*: c.189A>G (LB/VUS); *CA4*:c.700G>A (B)
19	F	53	8	OD 20/70–OS 20/200; PE OU	+	Family SMM	ND
20	F	31	1	OD 20/20–OS 20/20	+	Family SMM	ND—*RP1* sequencing
21	M	40	7	OD 20/25–OS 20/40; CAT OU	−	ND	*RP1*: c.6166G>A (LB/VUS); *SNRNP200*: c.1203+15G>A (LB); *USH2A*: c.12112A>G (VUS)
22	F	37	15	OD 20/25–OS 20/25; PE OU	+	Family SMM	ND
23	M	48	4	OD 20/20–OS 20/25; CAT OU	+	ND	*PROM1*:c.1345G>A (LB)
24	M	10	15	OD 20/100–OS 20/100	−	ND	*RP1*: c.788-12A>G (VUS)*PRPF6*: c.2638_2639delinsGC (VUS)*FSCN2*: c.619C>T (LB)
25	M	30	6	OD 20/25–OS 20/25; R-CME OU	+	Family CPL	ND
26	M	25	46	OD 20/200–OS HM	+	ND	ND—*RP1* sequencing
27	F	30	19	OD 20/20–OS 20/20	+	Family CR	ND
28	F	40	16	OD 20/20–OS 20/20; PRF	+	ND	ND

Abbreviation: B: benign. CAT: cataract. CD: corneal dystrophy. HM: hand motion. LB: likely benign. ND: not detected. OD: right eye. OS: left eye. OU: both eyes. P: pathogenic. PE: pseudophakic eye. PRF: preretinal fibrosis. Pt: patient. R-CME: regressed cystoid macular oedema. S-IOLO: secondary intraocular lens opacification. VUS: variant of uncertain significance. +: present. −: not present.

## Data Availability

Data regarding the clinical and genetic results of our patients are available from the different centres involved in the study, although protected by privacy regulations.

## Supplementary Materials

The following supporting information can be downloaded at https://www.mdpi.com/article/10.3390/medicina60020254/s1: NGS panels of RP/RCD-related tested genes at MAGI Euregio and AOOR Villa Sofia-Cervello.
