# Supplementary material for: RP1 Dominant p.Ser740* Pathogenic Variant in 20 Knowingly Unrelated Families Affected by Rod–Cone Dystrophy: Potential Founder Effect in Western Sicily
Source: Medicina (Kaunas). 2024 Feb 1;60(2):254. doi: 10.3390/medicina60020254 (PMC10890639; doi:10.3390/medicina60020254)
Supplement: Supplementary file 1 [file medicina-60-00254-s001.zip › RP1 Supplementary Material Content.pdf]

***RP1* Dominant p.Ser740* Pathogenic Variant In 20 Knowingly Unrelated Families Affected by Rod-Cone Dystrophy: Potential Founder Effect in Western Sicily**

D'Esposito F. et al.

Supplementary material content:

- SM1: Family CLB Pedigree
- SM2: Family SMM Pedigree
- SM3: Family CPL Pedigree
- SM4: Family CR Pedigree
- SM5: Patient #1 Pedigree
- SM6: Patient #2 Pedigree
- SM7: Patient #3 Pedigree
- SM8: Patient #10 Pedigree
- SM9: Patient #11 Pedigree
- SM10: Patient #12 Pedigree
- SM11: Patient #13 Pedigree
- SM12: Patient #14 Pedigree
- SM13: Patient #15 Pedigree
- SM14: Patient #16 Pedigree
- SM15: Patient #18 Pedigree
- SM16: Patient #21 Pedigree
- SM17: Patient #23 Pedigree
- SM18: Patient #24 Pedigree
- SM19: Patient #26 Pedigree
- SM20: Patient #28 Pedigree
- SM21: Caption to Pedigrees
- SM22: NGS Panels of RP-related genes tested in two centres
