# Supplementary material for: RP1 Dominant p.Ser740* Pathogenic Variant in 20 Knowingly Unrelated Families Affected by Rod–Cone Dystrophy: Potential Founder Effect in Western Sicily
Source: Medicina (Kaunas). 2024 Feb 1;60(2):254. doi: 10.3390/medicina60020254 (PMC10890639; doi:10.3390/medicina60020254)
Supplement: Supplementary file 1 [file medicina-60-00254-s001.zip › SM21 Caption to Pedigrees.pdf]

Caption to pedigrees

Pedigrees. Squared boxes indicate males, circles indicate females, closed symbols represent affected and open symbols represent unaffected persons. The arrows indicate the initial probands. Probands subsequently identified as being part of the same pedigree are indicated by the number assigned in this work. Double lines indicate consanguineous marriages. Question marks (?) indicate a possible but not proven affected status by relatives' interviewing. Pedigree of family CLB: patients nos. 4, 6, 7, 9 are indicated. Pedigree of family CR: patients nos. 5 and 27 are indicated. Pedigree of family CPL: patients nos. 8, 17, 25 are indicated. Pedigree of family SMM: patients nos. 19, 20, 22 are indicated.

Results of tested patients are shown. +/- indicates heterozygous individuals who carried the RP1 Ser740* variant. -/- indicates negative patients.
