# Supplementary material for: RP1 Dominant p.Ser740* Pathogenic Variant in 20 Knowingly Unrelated Families Affected by Rod–Cone Dystrophy: Potential Founder Effect in Western Sicily
Source: Medicina (Kaunas). 2024 Feb 1;60(2):254. doi: 10.3390/medicina60020254 (PMC10890639; doi:10.3390/medicina60020254)
Supplement: Supplementary file 1 [file medicina-60-00254-s001.zip › SM22 NGS panels of RP-related tested genes in two centres.pdf]

NGS panels of RP/RCD-related tested genes in two centres	
**MAGI Euregio, Bolzano, Italy**	**Ospedale Villa Sofia-Cervello, Palermo, Italy**
ABCA4 (OMIM #601691)	***ABCA4*** (OMIM #601691)
AGBL5 (OMIM #615900)	
AHI1 (OMIM #608894)	
ARHGEF18 (OMIM #616432)	
ARL2BP (OMIM #615407)	***ARL2BP*** (OMIM #615407)
ARL6 (OMIM #608845)	***ARL6*** (OMIM #608845)
BBS2 (OMIM #606151)	
BEST1 (OMIM #607854)	***BEST1*** (OMIM #607854)
PCARE (C2orf71) (OMIM #613425)	***PCARE (C2orf71)*** (OMIM #613425)
	CA4 (OMIM #114760)
CFAP410 *(C21orf2)* (OMIM #603191)	
C8orf37 (OMIM #614477)	***C8orf37*** (OMIM #614477)
CDHR1 (OMIM #609502)	
CERKL (OMIM #608381)	***CERKL*** (OMIM #608381)
CLRN1 (OMIM #606397)	***CLRN1*** (OMIM #606397)
CNGA1 (OMIM #123825)	***CNGA1*** (OMIM #123825)
CNGB1 (OMIM #600724)	***CNGB1*** (OMIM #600724)
CRB1 (OMIM #604210)	***CRB1*** (OMIM #604210)
CRX (OMIM #602225)	***CRX*** (OMIM #602225)
CYP4V2 (OMIM #608614)	***CYP4V2*** (OMIM #608614)
	DHX38 (OMIM #605584)
DHDDS (OMIM #608172)	***DHDDS*** (OMIM #608172)
	EMC1 (OMIM #616846)
EYS (OMIM #612424)	***EYS*** (OMIM #612424)
FAM161A (OMIM #613596)	***FAM161A*** (OMIM #613596)
FLVCR1 (OMIM #609144)	
	FSCN2 (OMIM #607643)
GUCA1B (OMIM #602275)	***GUCA1B*** (OMIM #602275)
	GPR125 (OMIM #612303)
HGSNAT (OMIM #610453)	
IDH3A (OMIM #601149)	
IDH3B (OMIM #604526)	***IDH3B*** (OMIM #604526)
IFT140 (OMIM #614620)	
IFT172 (OMIM #607386)	
IMPDH1 (OMIM #146690)	***IMPDH1*** (OMIM #146690)
IMPG2 (OMIM #607056)	***IMPG2*** (OMIM #607056)
	KIAA1549 (MIM #613344)
KIZ (OMIM #615757)	***KIZ*** (OMIM #615757)
KLHL7 (OMIM #611119)	***KLHL7*** (OMIM #611119)
LRAT (OMIM #604863)	***LRAT*** (OMIM #604863)
MAK (OMIM #154235)	***MAK*** (OMIM #154235)
MERTK (OMIM #604705)	***MERTK*** (OMIM #604705)
	MVK (OMIM #610377)
	NEK2 (OMIM #604043)
	NPHP4 (OMIM #606996)
NR2E3 (OMIM #604485)	***NR2E3*** (OMIM #604485)
NRL (OMIM #162080)	***NRL*** (OMIM #162080)
OFD1 (OMIM #300170)	***OFD1*** (OMIM #300170)
PDE6A (OMIM #180071)	***PDE6A*** (OMIM #180071)
PDE6B (OMIM #180072)	***PDE6B*** (OMIM #180072)

PDE6G (OMIM #180073)	***PDE6G*** (OMIM #180073)
POMGNT1 (OMIM #606822)	
PRCD (OMIM #610598)	***PRCD*** (OMIM #610598)
PROM1 (OMIM #604365)	***PROM1*** (OMIM #604365)
PRPF3 (OMIM #607301)	***PRPF3*** (OMIM #607301)
PRPF4 (OMIM #607795)	***PRPF4*** (OMIM #607795)
PRPF6 (OMIM #613979)	***PRPF6*** (OMIM #613979)
PRPF8 (OMIM #607300)	***PRPF8*** (OMIM #607300)
PRPF31 (OMIM #606419)	***PRPF31*** (OMIM #606419)
PRPH2 (OMIM #179605)	***PRPH2*** (OMIM #179605)
	RBP3 (OMIM #180290)
RDH12 (OMIM #608830)	***RDH12*** (OMIM #608830)
REEP6 (OMIM #609346)	
RGR (OMIM #600342)	***RGR*** (OMIM #600342)
RHO (OMIM #180380)	***RHO*** (OMIM #180380)
RLBP1 (OMIM #180090)	***RLBP1*** (OMIM #180090)
ROM1 (OMIM #180721)	***ROM1*** (OMIM #180721)
RP1 (OMIM #603937)	***RP1*** (OMIM #603937)
RP1L1 (OMIM #608581)	***RP1L1*** (OMIM #608581)
RP2 (OMIM #300757)	***RP2*** (OMIM #300757)
RP9 (OMIM #607331)	***RP9*** (OMIM #607331)
RPE65 (OMIM #180069)	***RPE65*** (OMIM #180069)
RPGR (OMIM #312610)	***RPGR*** (OMIM #312610)
	RRH (OMIM #605224)
SAG (OMIM #181031)	***SAG*** (OMIM #181031)
SEMA4A (OMIM #607292)	***SEMA4A*** (OMIM #607292)
SLC7A14 (OMIM #615720)	***SLC7A14*** (OMIM #615720)
SNRNP200 (OMIM #601664)	***SNRNP200*** (OMIM #601664)
SPATA7 (OMIM #609868)	***SPATA7*** (OMIM #609868)
TOPORS (OMIM #609507)	***TOPORS*** (OMIM #609507)
TTC8 (OMIM #608132)	***TTC8*** (OMIM #608132)
TULP1 (OMIM #602280)	***TULP1*** (OMIM #602280)
USH2A (OMIM #608400)	***USH2A*** (OMIM #608400)
ZNF408 (OMIM #616454)	
