# Supplementary figures and images for: RP1 Dominant p.Ser740* Pathogenic Variant in 20 Knowingly Unrelated Families Affected by Rod–Cone Dystrophy: Potential Founder Effect in Western Sicily
Source: Medicina (Kaunas). 2024 Feb 1;60(2):254. doi: 10.3390/medicina60020254 (PMC10890639; doi:10.3390/medicina60020254)

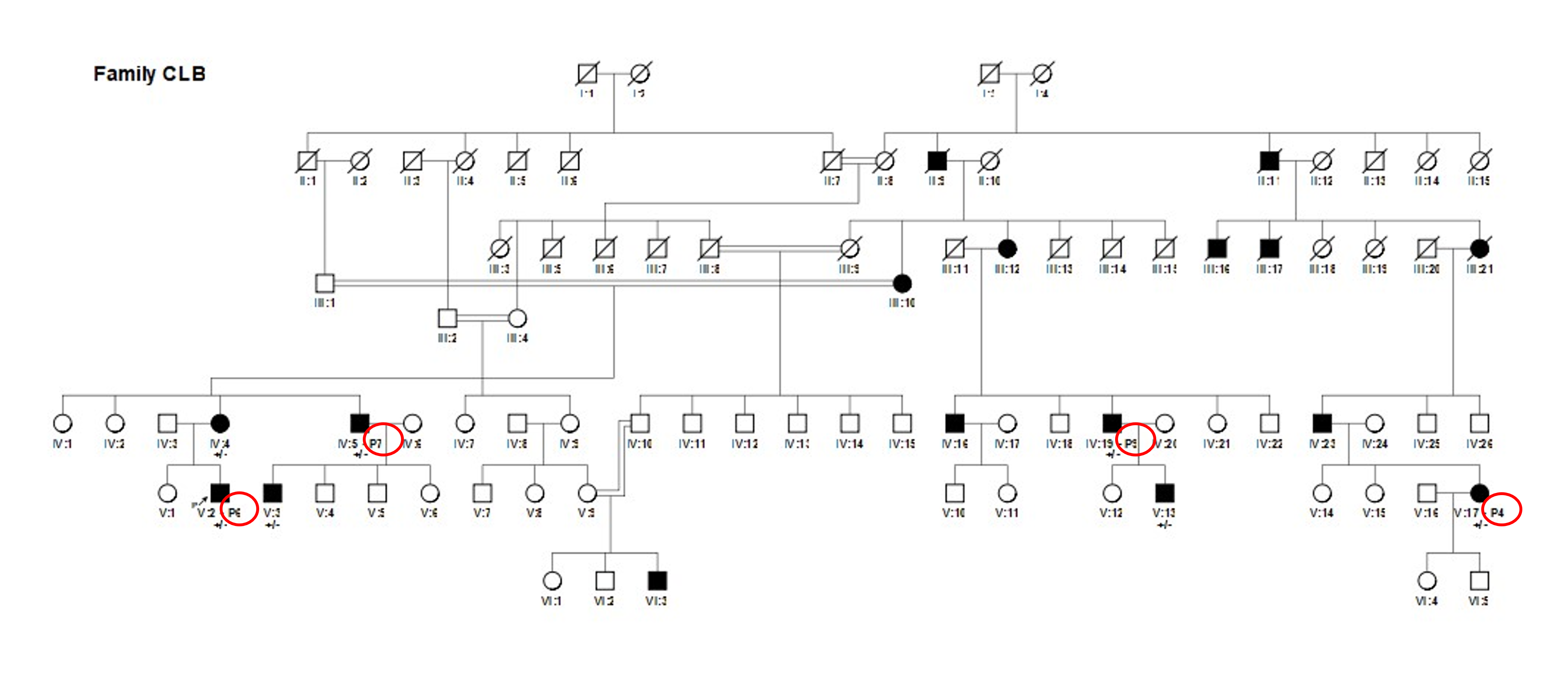

Supplement: Supplementary file 1 [file medicina-60-00254-s001.zip › SM1 CLB Family Pedigree.png]

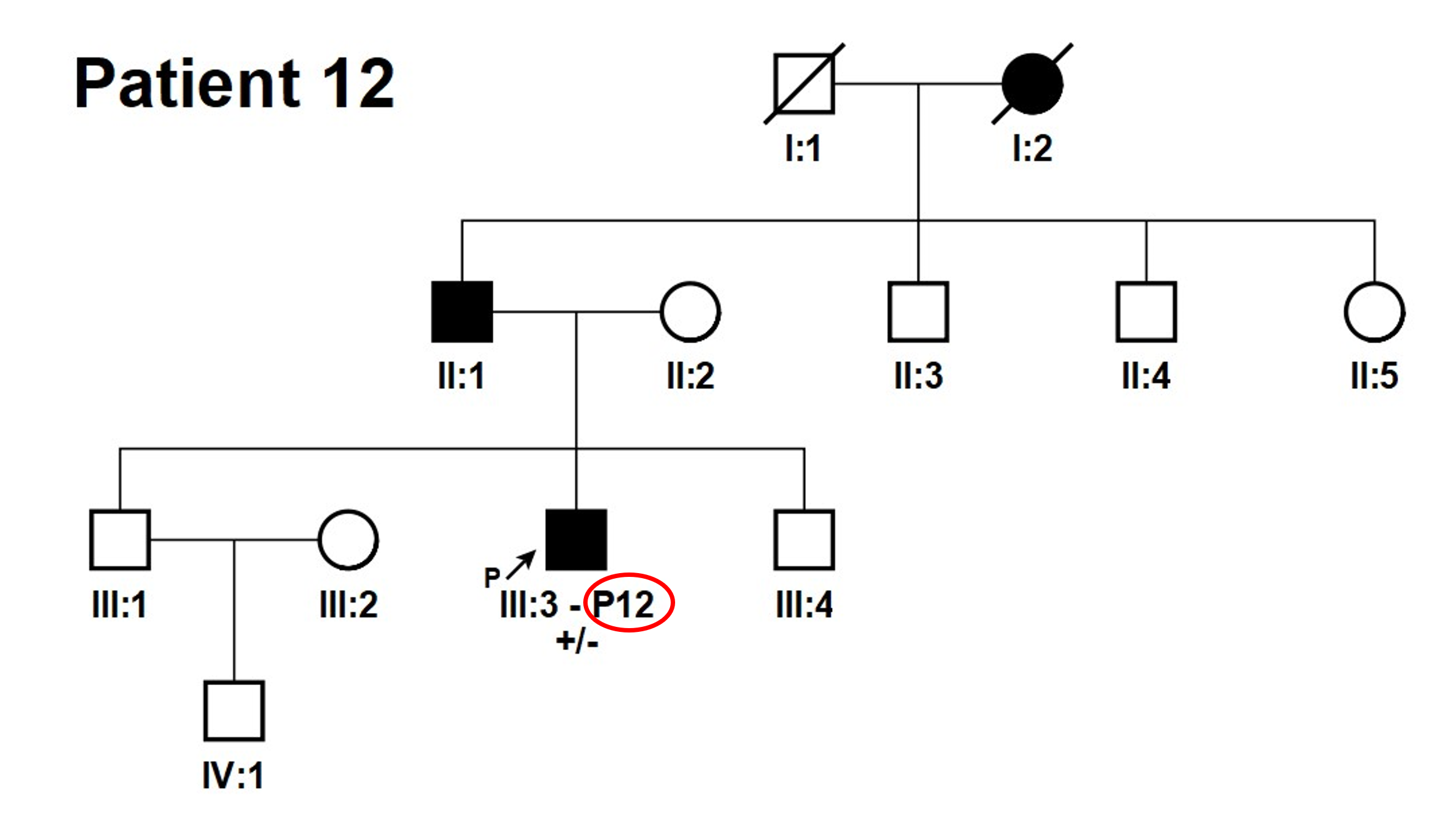

Supplement: Supplementary file 1 [file medicina-60-00254-s001.zip › SM10 Patient 12 Pedigree.png]

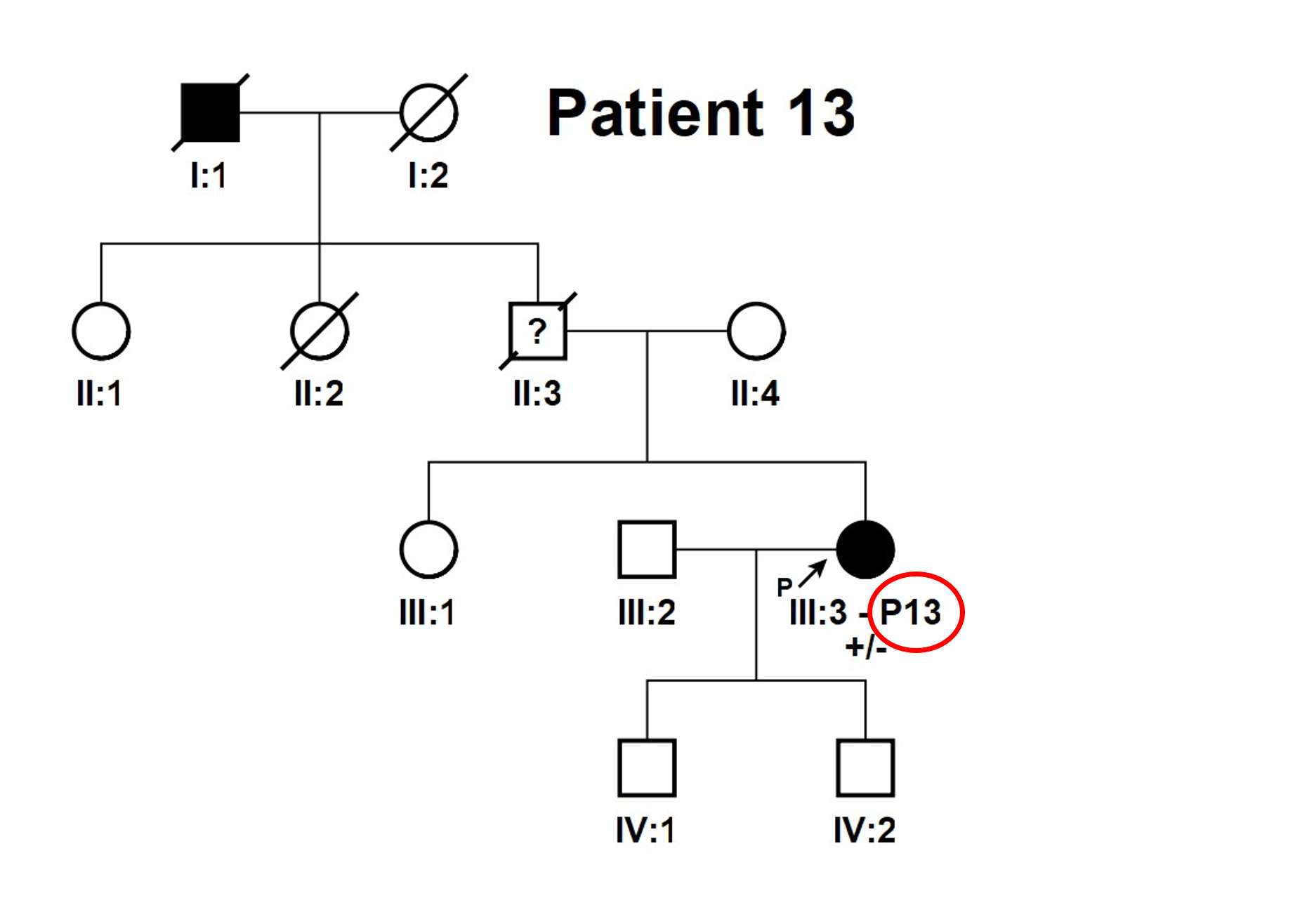

Supplement: Supplementary file 1 [file medicina-60-00254-s001.zip › SM11 Patient 13 Pedigree.png]

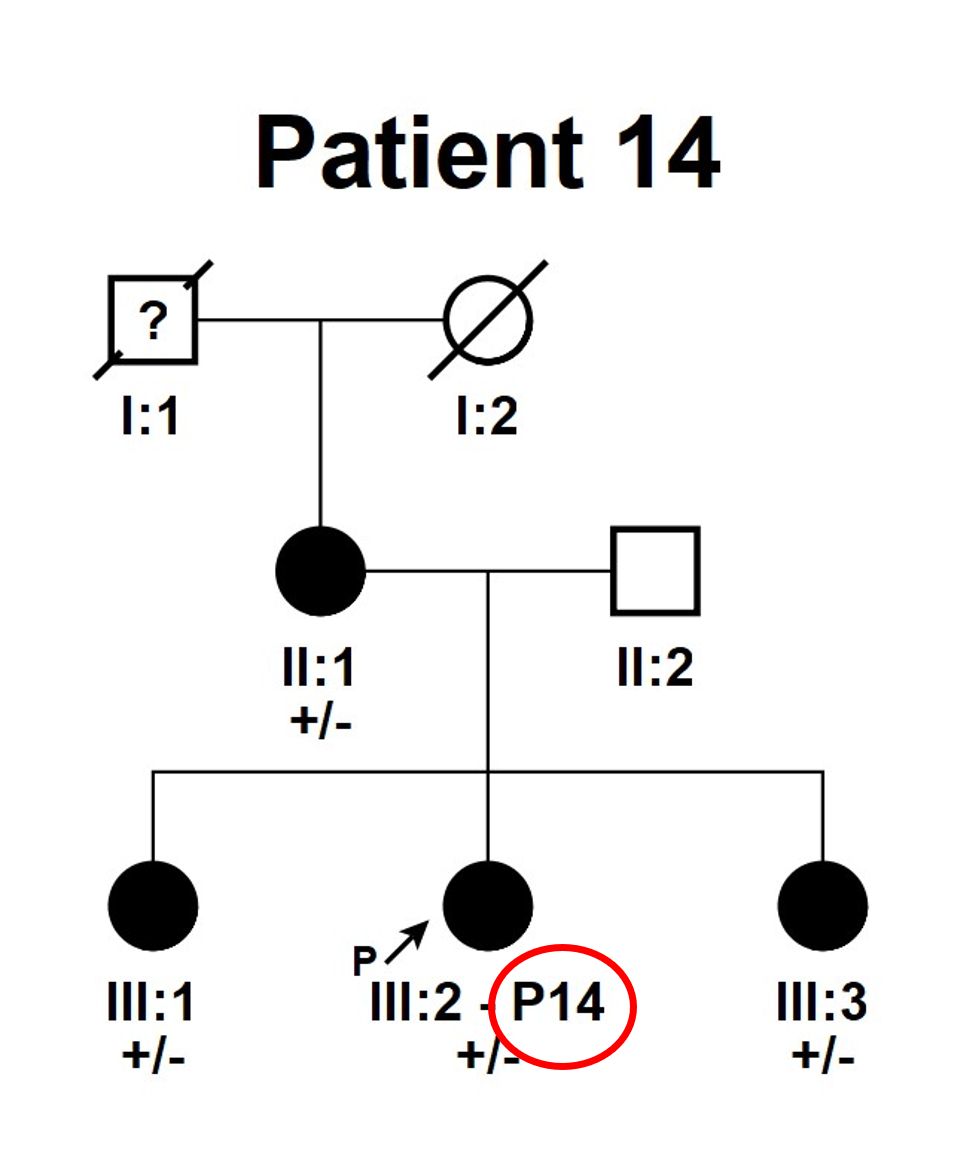

Supplement: Supplementary file 1 [file medicina-60-00254-s001.zip › SM12 Patient 14 Pedigree.png]

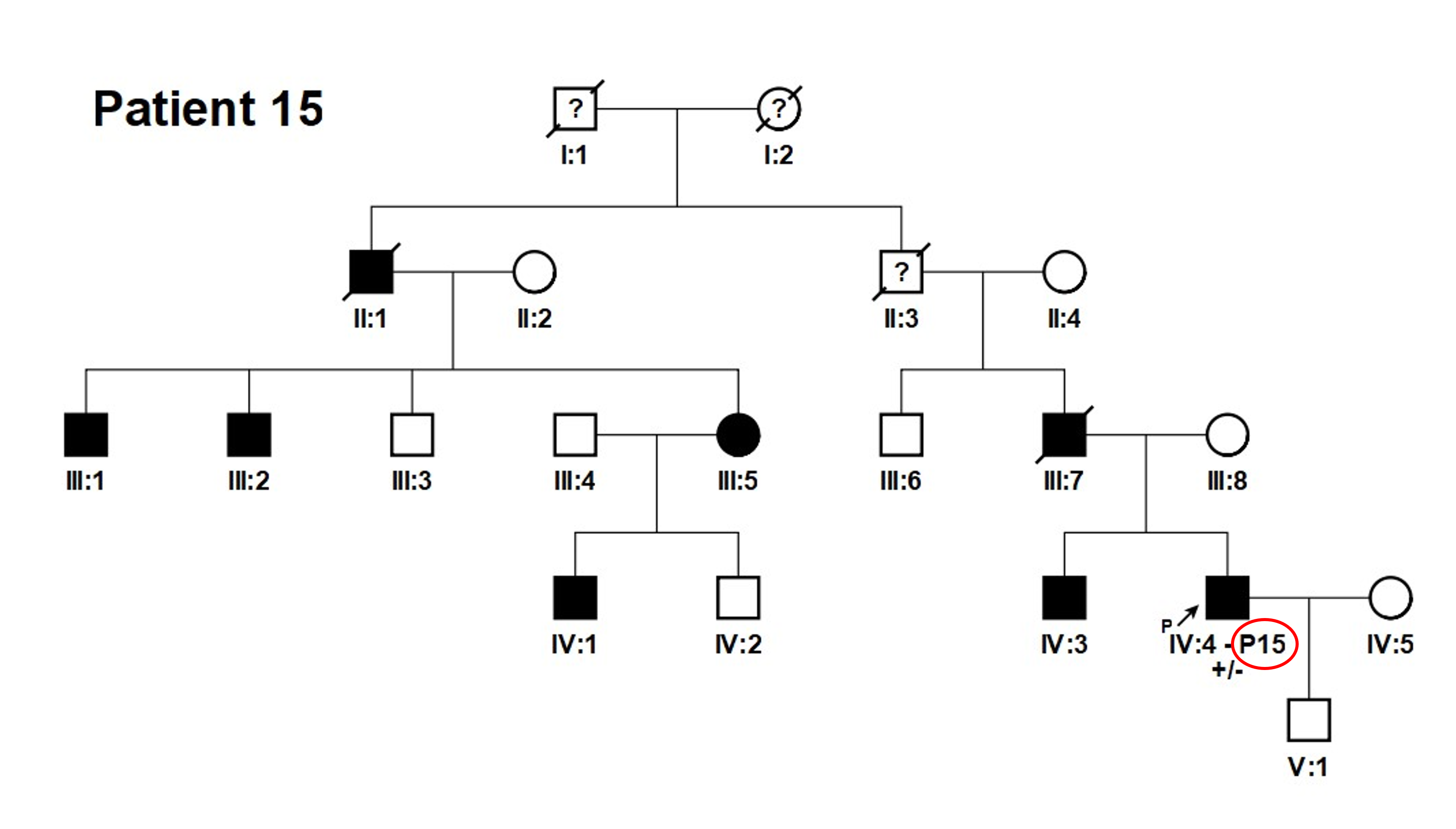

Supplement: Supplementary file 1 [file medicina-60-00254-s001.zip › SM13 Patient 15 Pedigree.png]

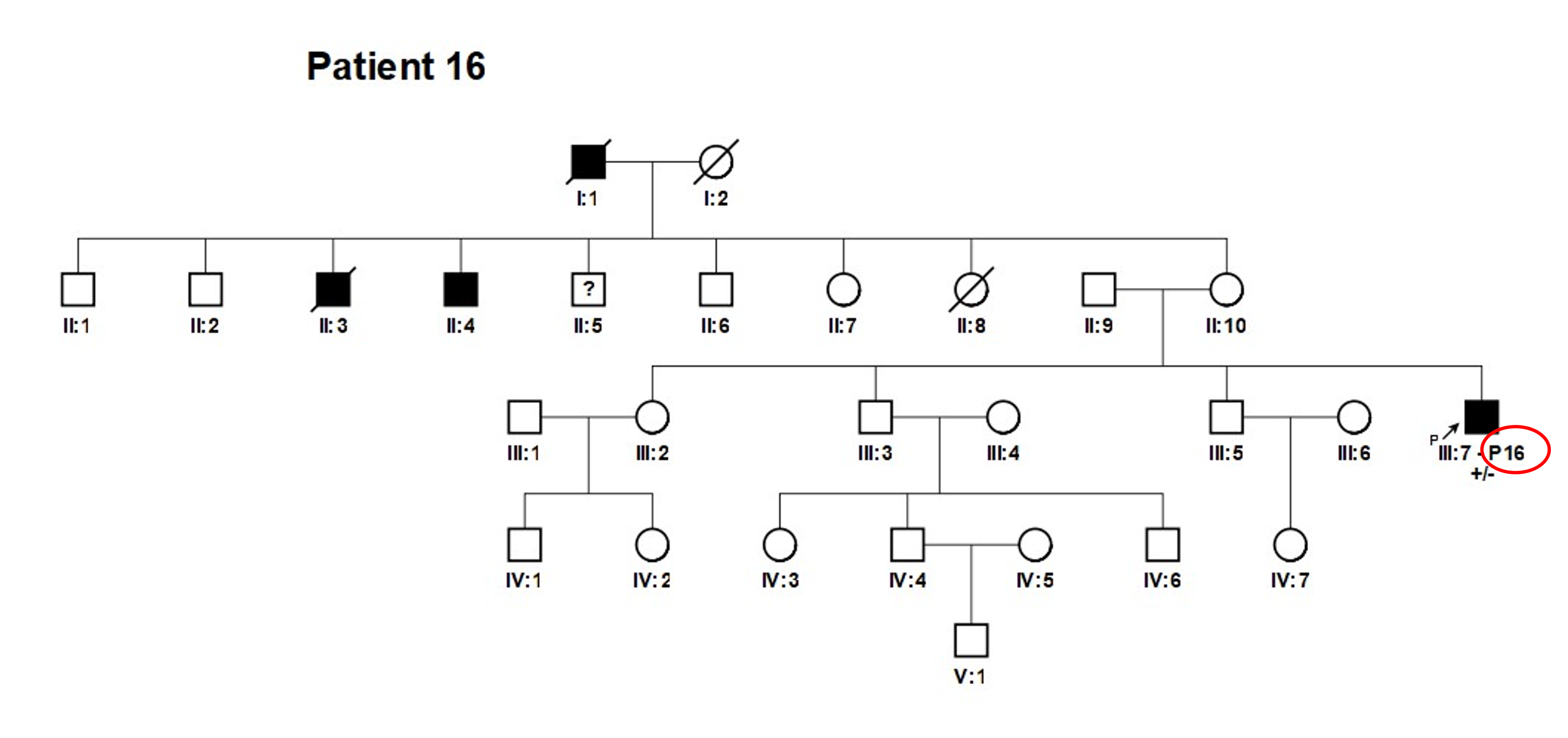

Supplement: Supplementary file 1 [file medicina-60-00254-s001.zip › SM14 Patient 16 Pedigree.png]

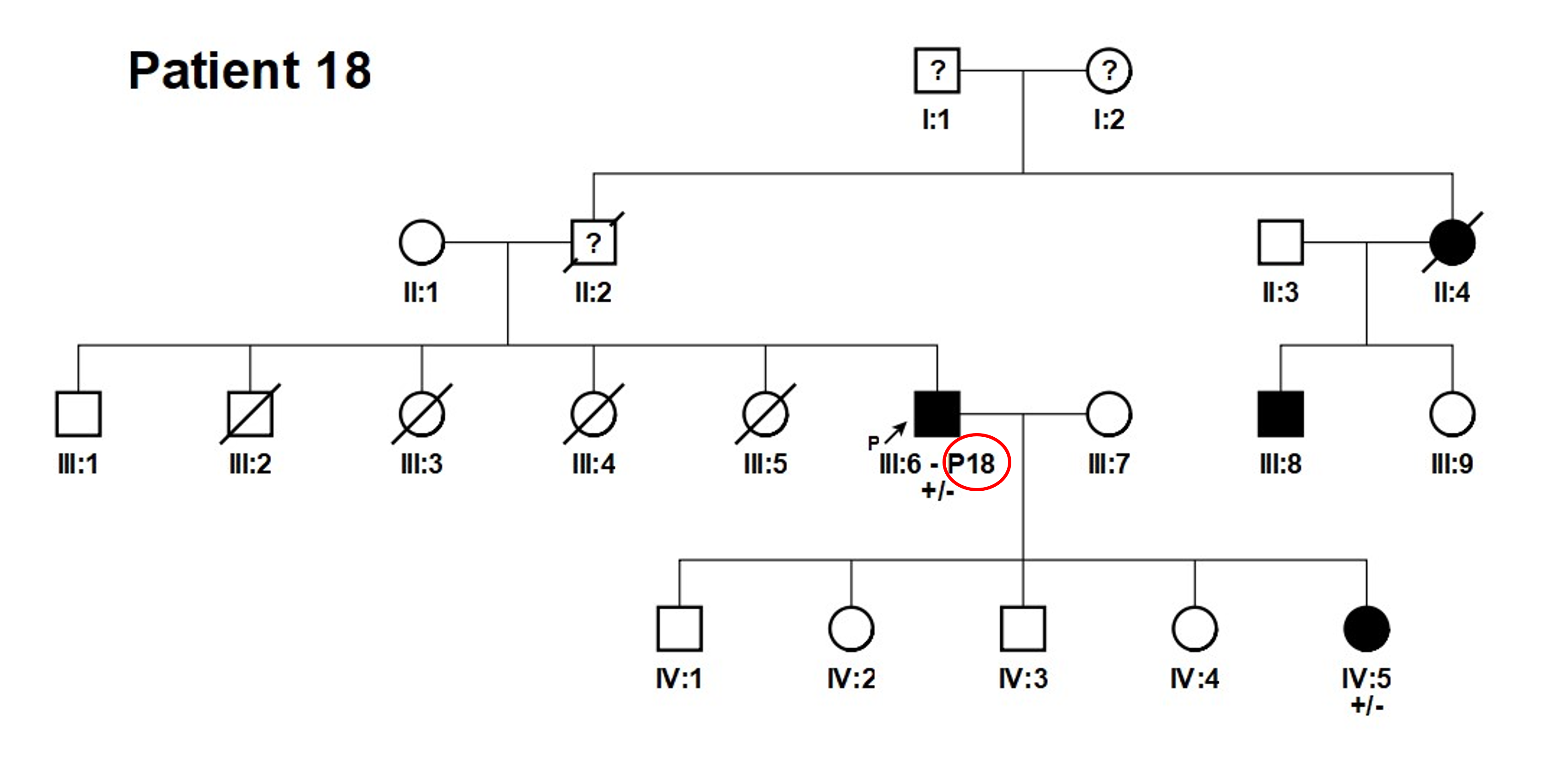

Supplement: Supplementary file 1 [file medicina-60-00254-s001.zip › SM15 Patient 18 Pedigree.png]

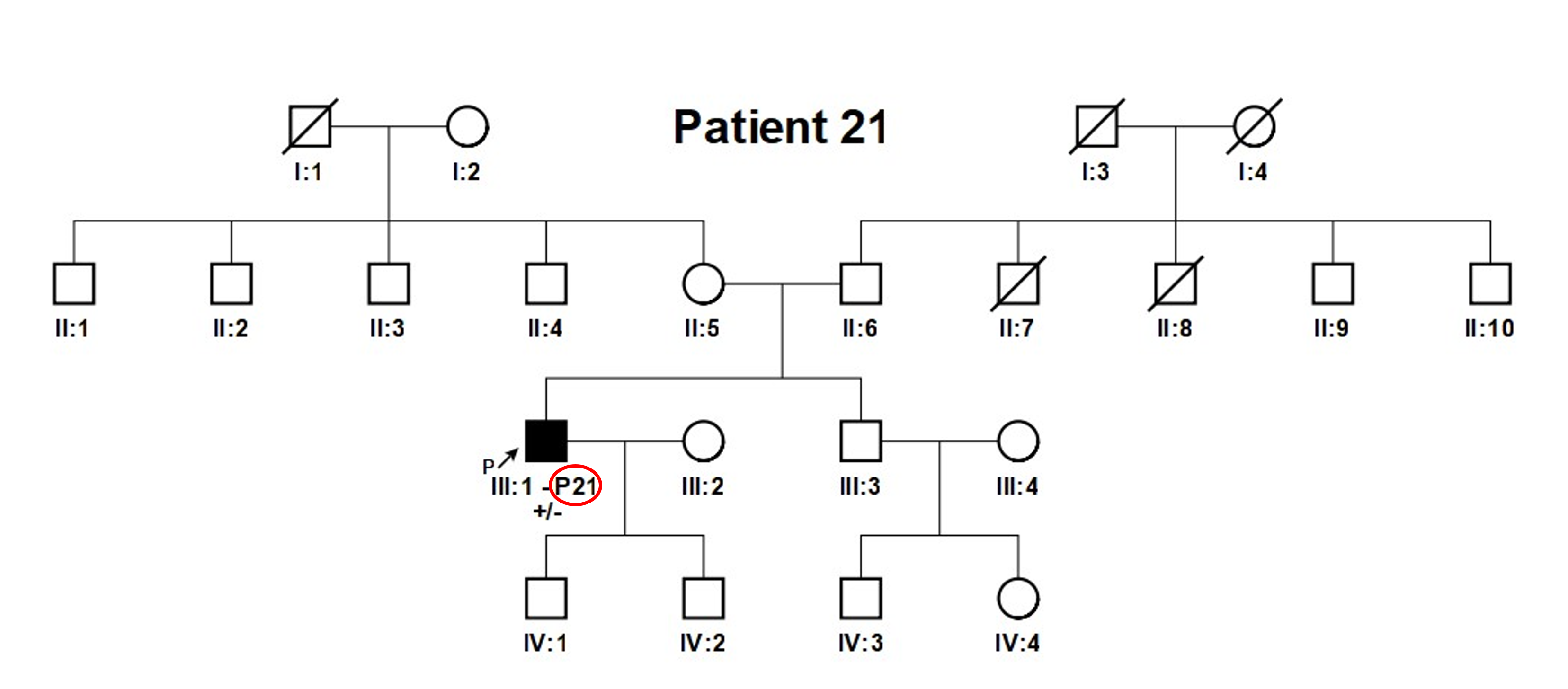

Supplement: Supplementary file 1 [file medicina-60-00254-s001.zip › SM16 Patient 21 Pedigree.png]

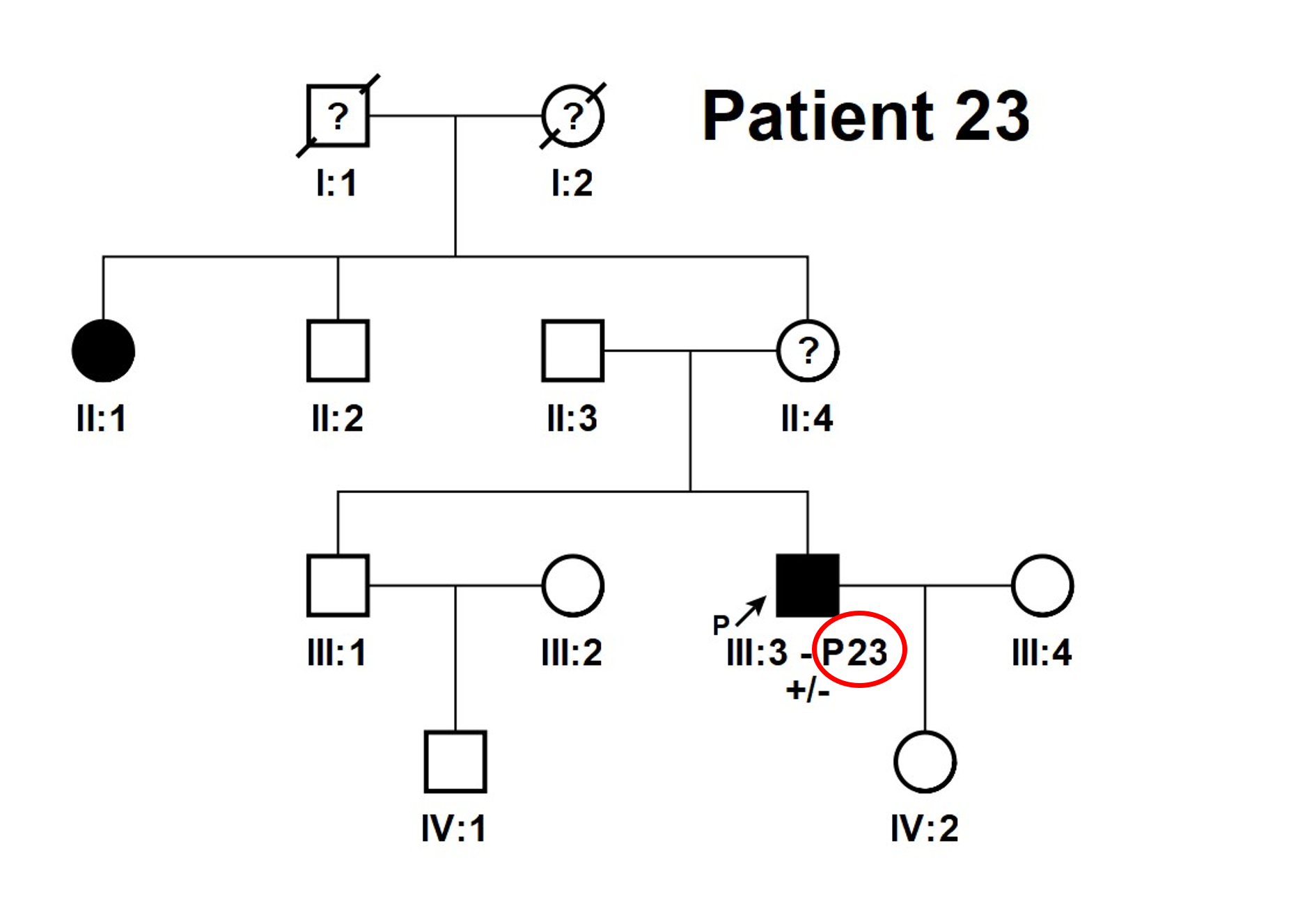

Supplement: Supplementary file 1 [file medicina-60-00254-s001.zip › SM17 Patient 23 Pedigree.png]

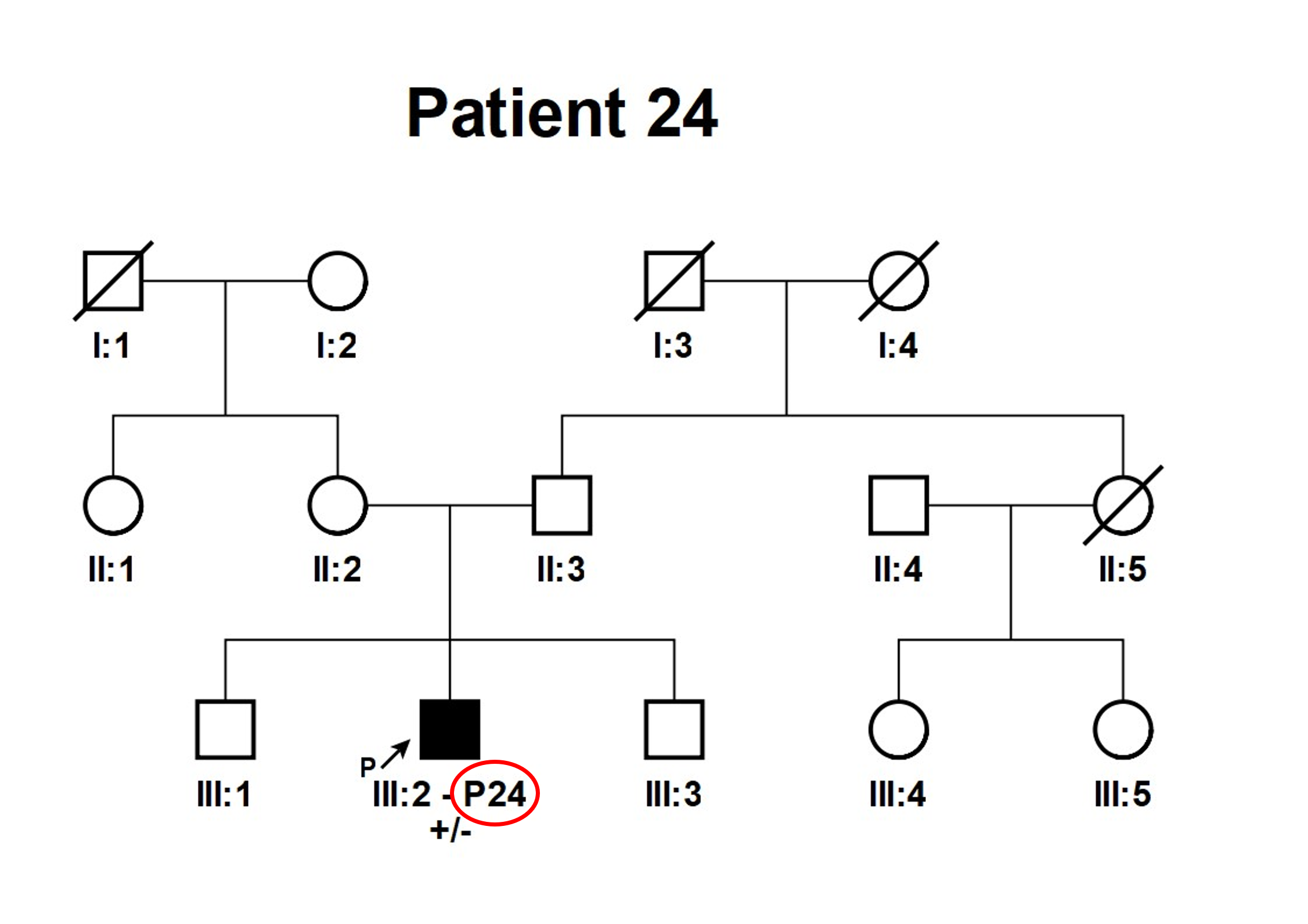

Supplement: Supplementary file 1 [file medicina-60-00254-s001.zip › SM18 Patient 24 Pedigree.png]

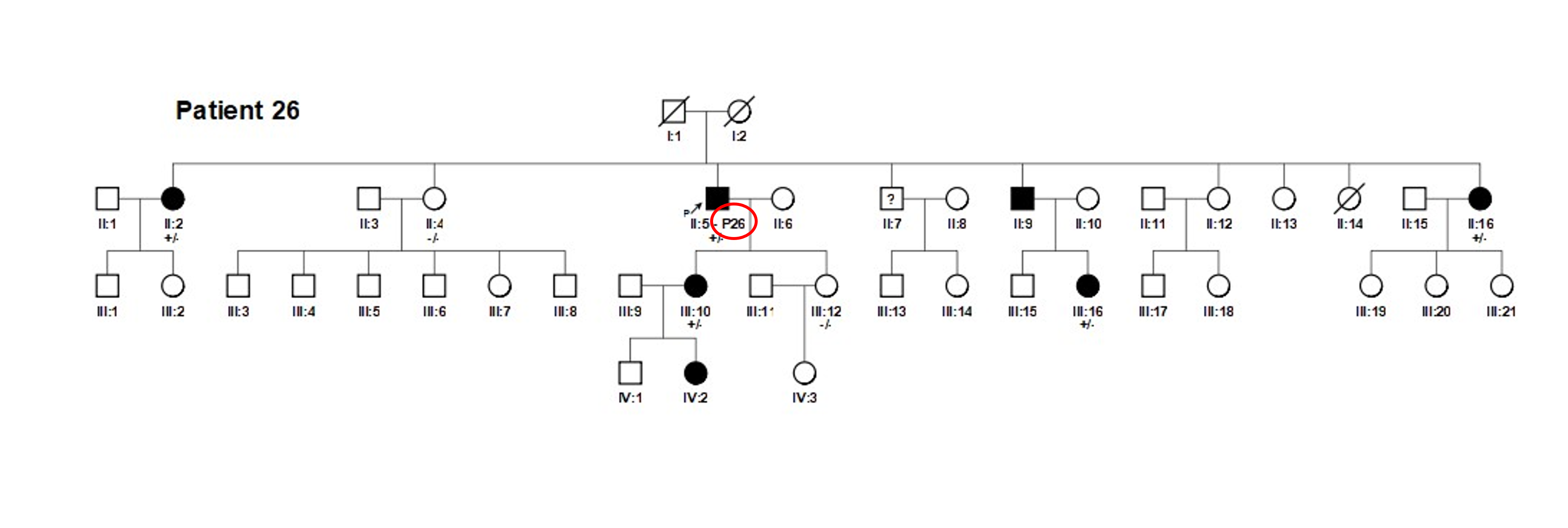

Supplement: Supplementary file 1 [file medicina-60-00254-s001.zip › SM19 Patient 26 Pedigree.png]

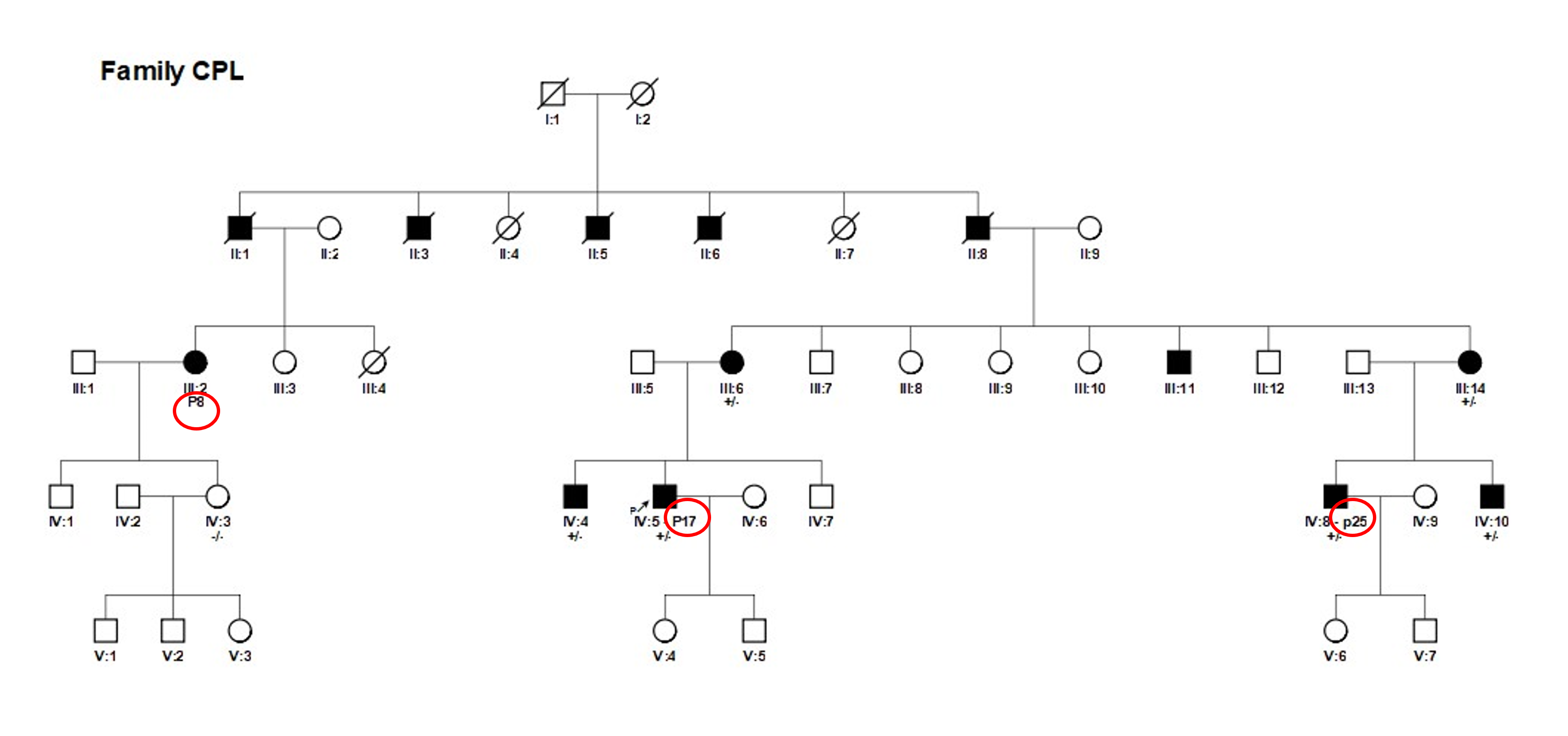

Supplement: Supplementary file 1 [file medicina-60-00254-s001.zip › SM2 CPL Family Pedigree.png]

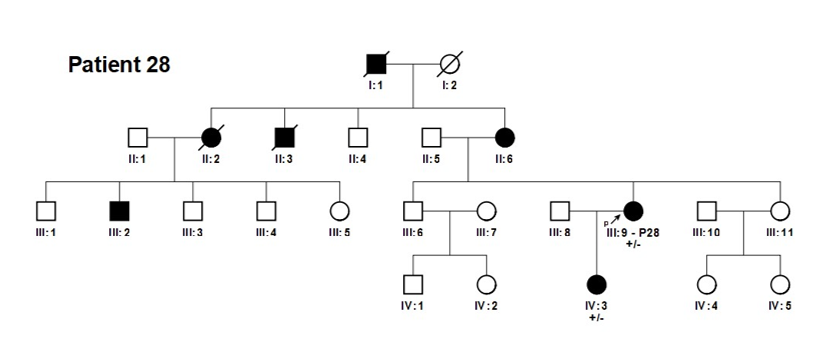

Supplement: Supplementary file 1 [file medicina-60-00254-s001.zip › SM20 Patient 28 Pedigree.png]

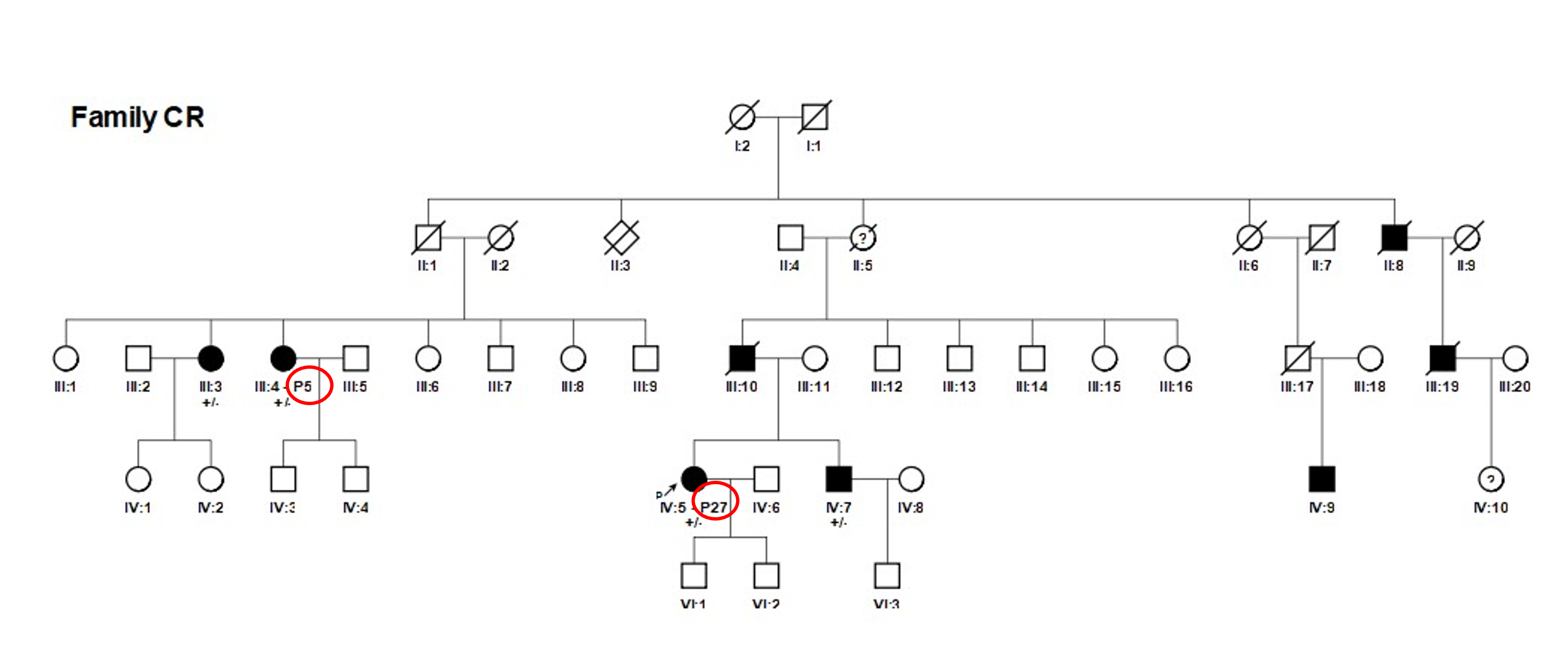

Supplement: Supplementary file 1 [file medicina-60-00254-s001.zip › SM3 CR Family Pedigree.png]

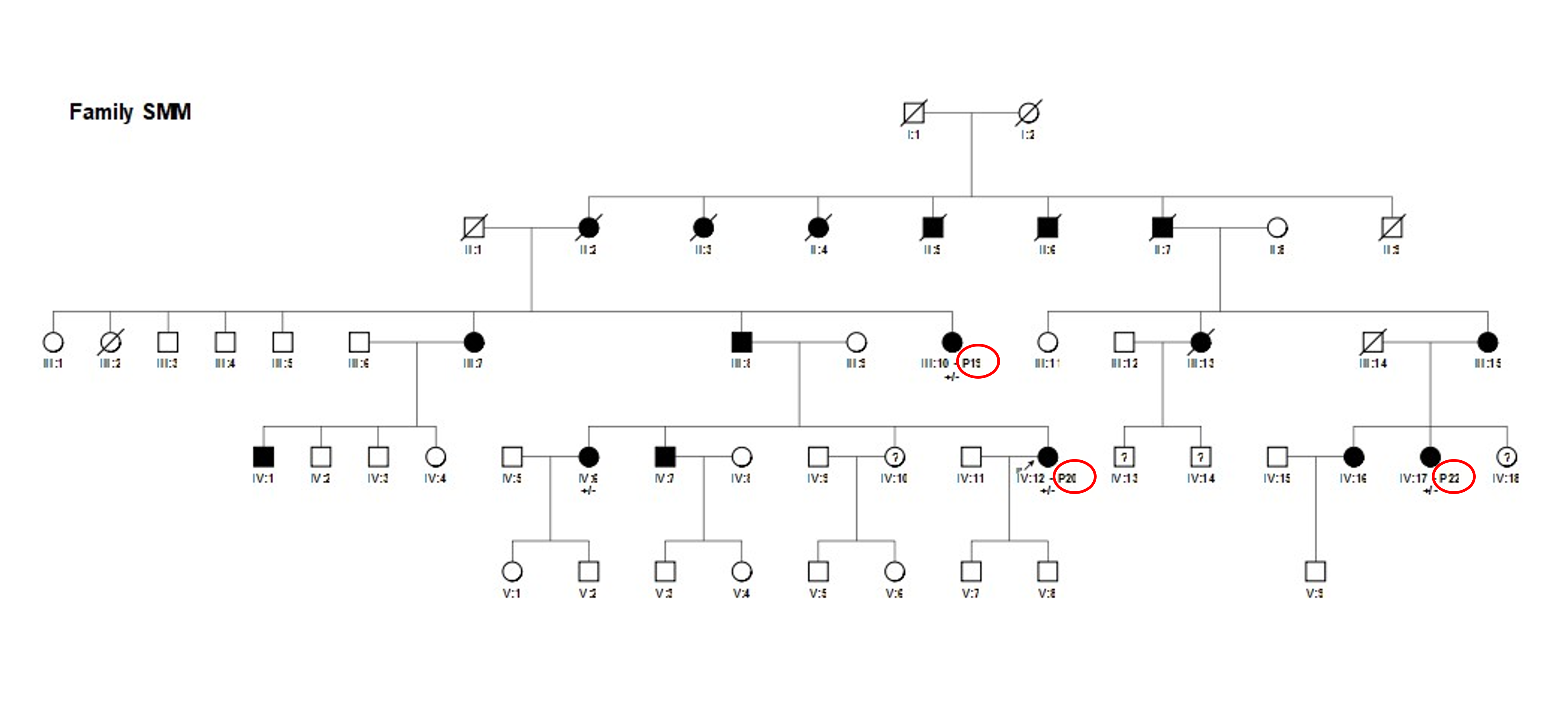

Supplement: Supplementary file 1 [file medicina-60-00254-s001.zip › SM4 SMM Family Pedigree.png]

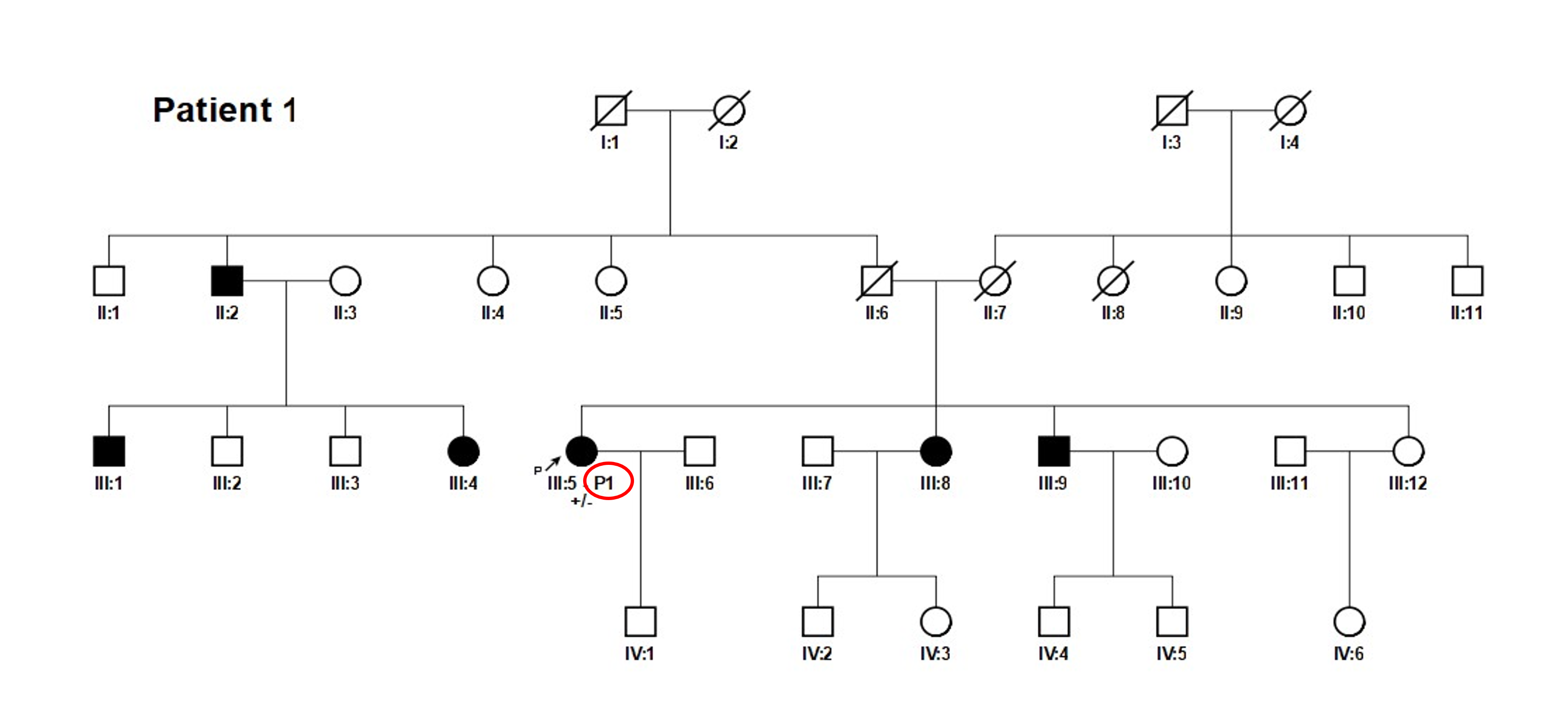

Supplement: Supplementary file 1 [file medicina-60-00254-s001.zip › SM5 Patient 1 Pedigree.png]

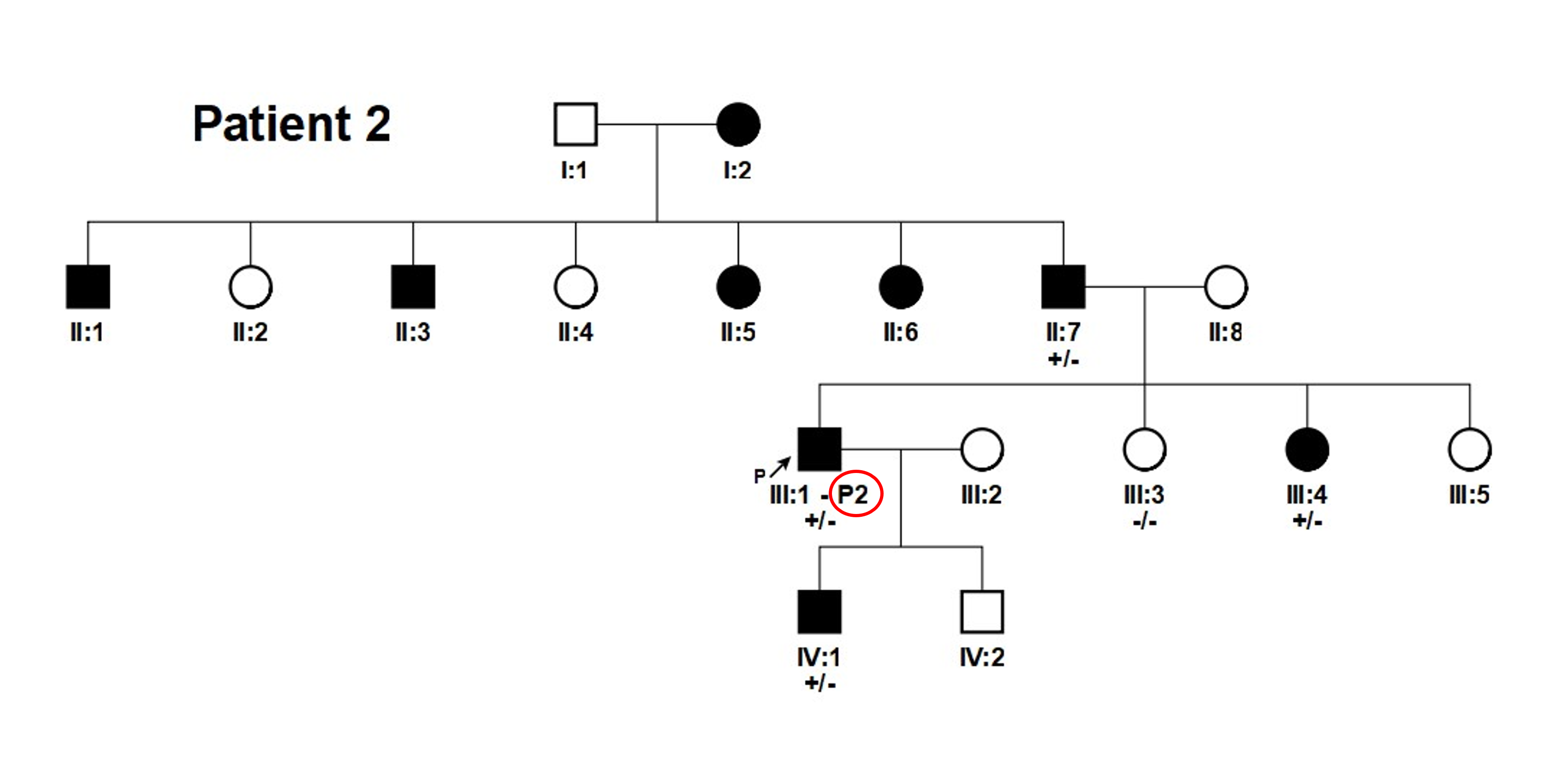

Supplement: Supplementary file 1 [file medicina-60-00254-s001.zip › SM6 Patient 2 Pedigree.png]

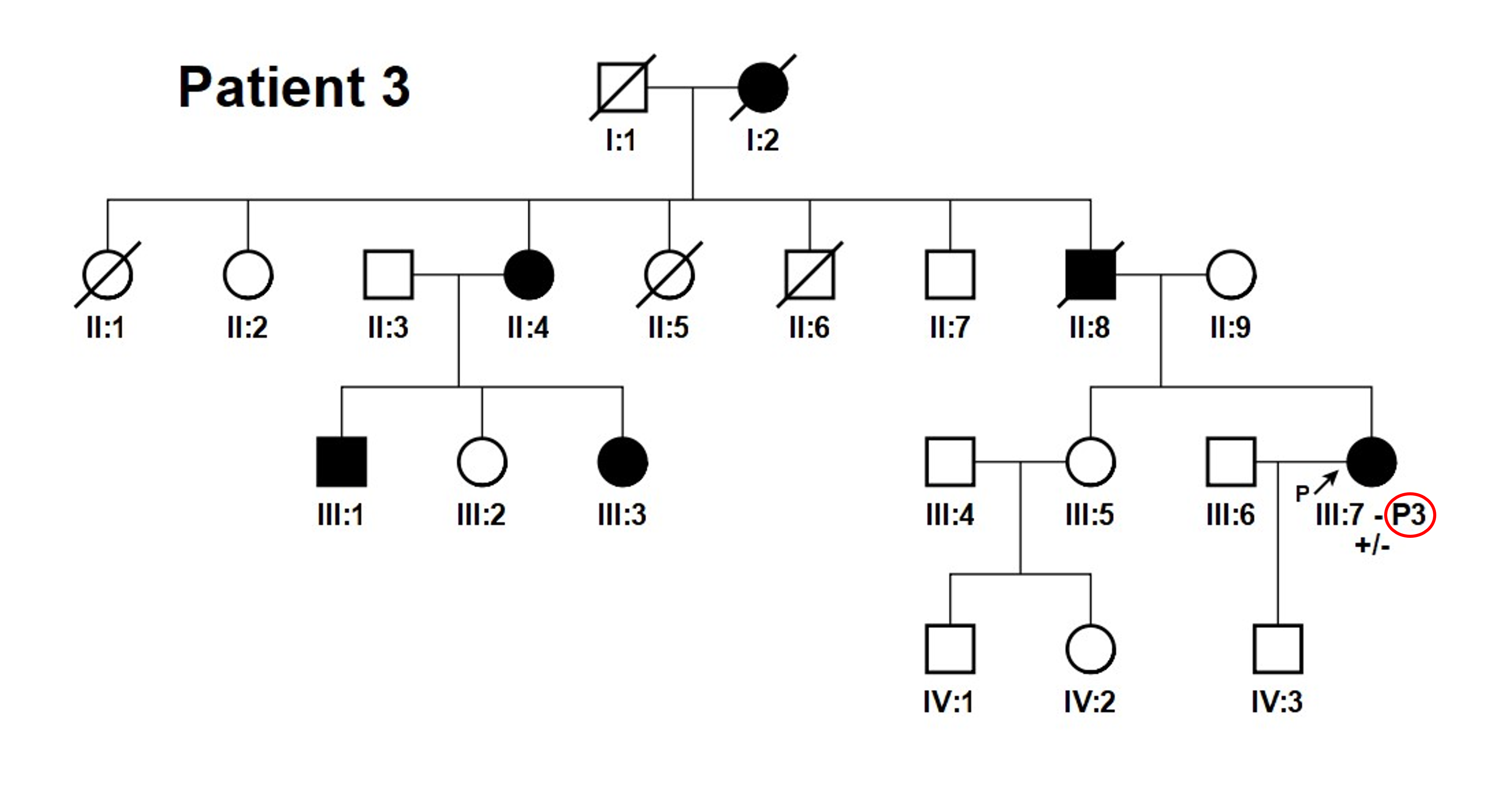

Supplement: Supplementary file 1 [file medicina-60-00254-s001.zip › SM7 Patient 3 Pedigree.png]

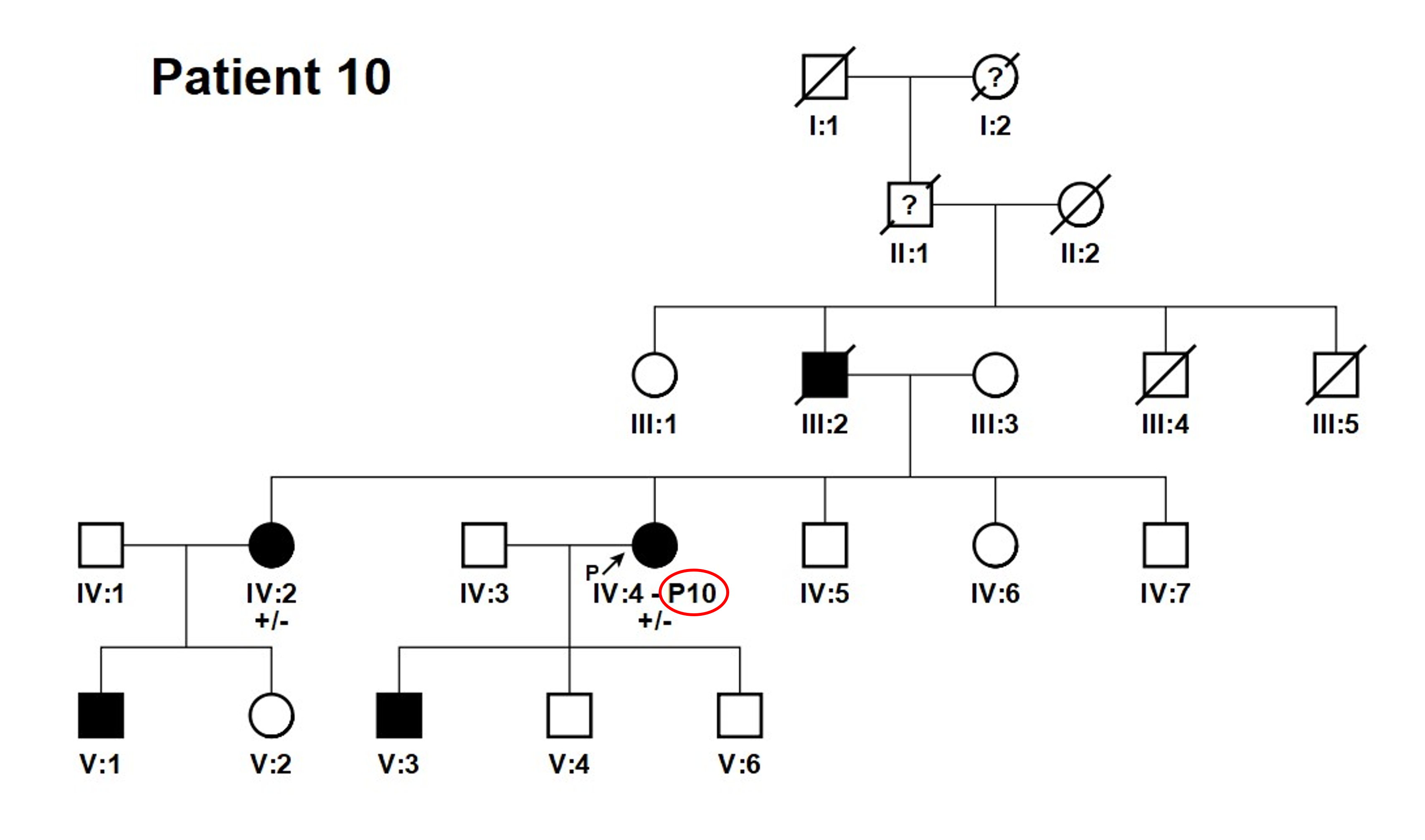

Supplement: Supplementary file 1 [file medicina-60-00254-s001.zip › SM8 Patient 10 Pedigree.png]

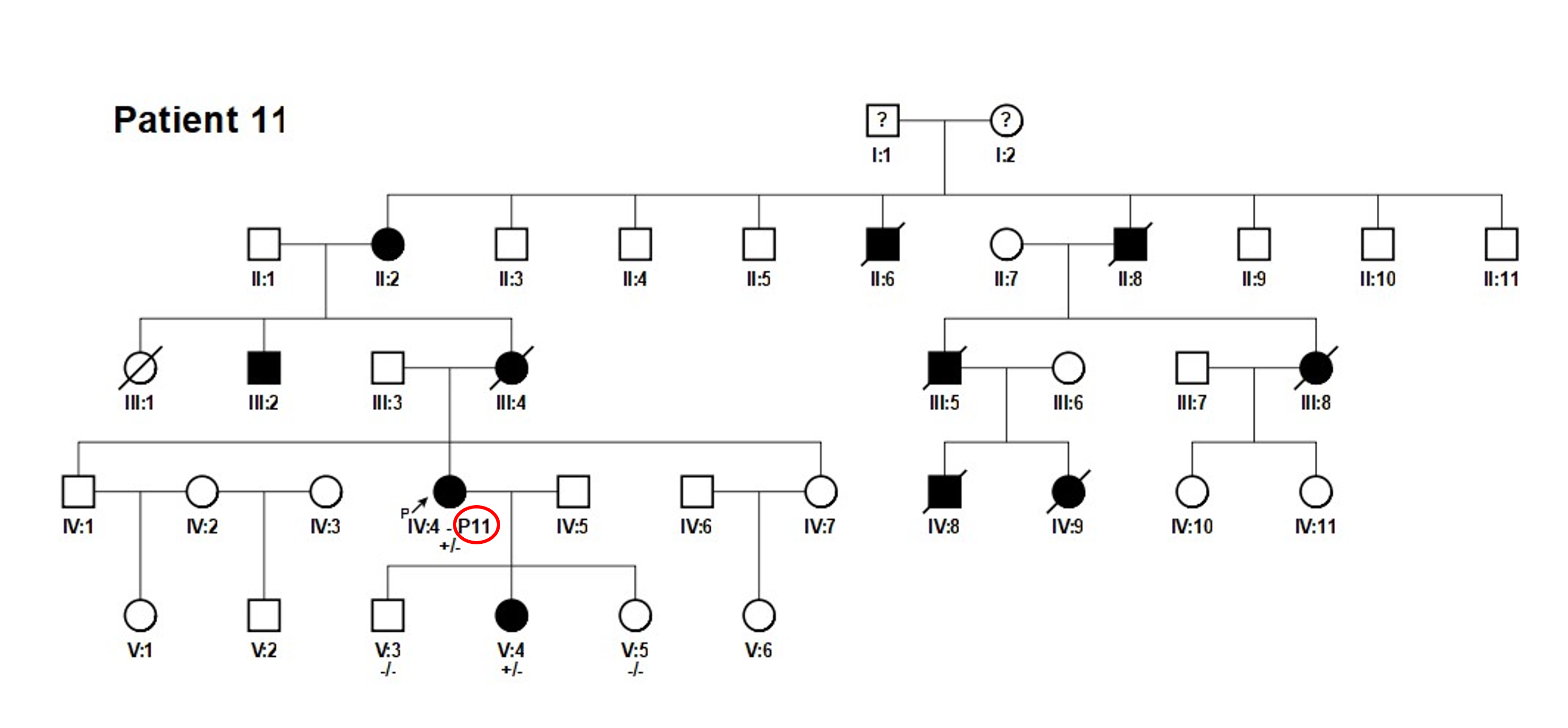

Supplement: Supplementary file 1 [file medicina-60-00254-s001.zip › SM9 Patient 11 Pedigree.png]
